# SPD_0090 Negatively Contributes to Virulence of *Streptococcus pneumoniae*

**DOI:** 10.3389/fmicb.2022.896896

**Published:** 2022-06-13

**Authors:** Linlin Cao, Nan Li, Yingshan Dong, Xiao-Yan Yang, Jiajia Liu, Qing-Yu He, Ruiguang Ge, Xuesong Sun

**Affiliations:** ^1^MOE Key Laboratory of Tumor Molecular Biology and Key Laboratory of Functional Protein Research of Guangdong Higher Education Institutes, Institute of Life and Health Engineering, College of Life Science and Technology, Jinan University, Guangzhou, China; ^2^State Key Laboratory of Biocontrol, School of Life Sciences, Sun Yat-sen University, Guangzhou, China

**Keywords:** *Streptococcus pneumoniae*, iron uptake, ABC transporter, virulence, infection

## Abstract

In most bacteria, iron plays an important role in the survival of bacteria and the process of infection to the host. *Streptococcus pneumoniae* (*S. pneumoniae*) evolved three iron transporters (i.e., PiaABC, PiuABC, and PitABC) responsible for the transportation of three kinds of iron (i.e., ferrichrome, hemin, and ferric ion). Our previous study showed that both mRNA and protein levels of SPD_0090 were significantly upregulated in the Δ*piuA/*Δ*piaA/*Δ*pitA* triple mutant, but its detailed biological function is unknown. In this study, we constructed *spd*_*0090* knockout and complement strain and found that the deletion of *spd_0090* hinders bacterial growth. SPD_0090 is located on the cell membrane and affects the hemin utilization ability of *S. pneumoniae*. The cell infection model showed that the knockout strain had stronger invasion and adhesion ability. Notably, knockout of the *spd*_*0090* gene resulted in an enhanced infection ability of *S. pneumoniae* in mice by increasing the expression of virulence factors. Furthermore, iTRAQ quantitative proteomics studies showed that the knockout of *spd_0090* inhibited carbon metabolism and thus suppressed bacterial growth. Our study showed that SPD_0090 negatively regulates the virulence of *S. pneumoniae.*

## Introduction

*Streptococcus pneumoniae* (*S. pneumoniae*) is one of the bacteria frequently causing fatal infections (Bogaert et al., 2004; Engholm et al., 2017). Young children, the elderly, and patients with immunodeficiency are particularly susceptible to pneumococcal infections. An estimated 14.5 million children are infected with *S. pneumoniae* each year, two-thirds of which occur in Asia or Africa. Therefore, the World Health Organization considers *S. pneumoniae* to be a priority bacterium requiring urgent development of novel antibiotics or anti-infective strategies (Kadioglu et al., 2008; Weight et al., 2021).

To survive and infect the host, *S. pneumoniae* must obtain metal ions (e.g., iron, manganese, zinc, and copper) (Honsa et al., 2013). Iron is an essential micronutrient for eukaryotes and prokaryotes as it participates in several key metabolic processes, such as amino acid synthesis, tricarboxylic acid cycle activity (TCA), DNA replication, cell respiration, and electron transport (Sheldon and Heinrichs, 2015; Burcham et al., 2020). As most iron is bound to glycoproteins (lactoferrin) and hemin-containing proteins (hemoglobin), the amount of free iron ions available in the human host is extremely limited. To support survival and infection, *S. pneumoniae* has evolved three ABC-transport proteins, namely, PiaABC, PiuABC, and PitABC, of which the lipoproteins PiaA, PiuA, and PitA serve as substrate-binding proteins for iron uptake (Brown et al., 2001a,^2002^). In recent years, lipoproteins SPD_1609 and SPD_1590 have also been found to transport iron in our group (Miao et al., 2018; Yang et al., 2019). The ability of iron acquisition under physiological conditions contributes to bacterial virulence. For example, PiaA and PiuA have been shown to serve as virulence factors necessary for *S. pneumoniae* infection in mice (Payne and Neilands, 1988; Jomaa et al., 2005; Gupta et al., 2009).

Our previous study has shown that a Δ*piuA/*Δ*piaA/*Δ*pitA* triple mutant is able to grow, albeit slowly, in an iron-containing medium, which suggests that there are likely to be additional iron-uptake systems in *S. pneumoniae*. SPD_0090 was highly expressed at protein levels in the triple mutant strain (D39Δ*piaA/*Δ*piuA/*Δ*pitA*), implying that it is possibly involved in iron transport (Yang et al., 2016). Therefore, we constructed *spd*_*0090* knockout and complement strains, and *in vitro* expressed SPD_0090 protein, which was characterized by *in vivo* and *in vitro* biochemical experiments. Moreover, quantitative proteomics was performed to find out the role of *spd*_*0090* gene deletion in maintaining *S. pneumoniae* metabolism and infection. The contribution of SPD_0090 to the virulence uncovered by this study will provide important information for a deep understanding of the pathogenic mechanisms of *S. pneumoniae*.

## Materials and Methods

### Sequence Analysis

The protein sequence of SPD_0090 from *S. pneumoniae* D39 was used as a seed to query the UniProt database using the Protein BLAST tool. Then, multiple sequence alignment and cluster analysis were performed with high-scoring proteins using the software package Clustal-X 2.1 (Zheng et al., 2021).

### Growth Media and Culture Conditions of Bacterial Strains

*S. pneumoniae* D39 wild type (WT) was cultured in THY medium consisting of Todd-Hewitt broth (Oxoid, United Kingdom) supplemented with 0.5% yeast extract (Oxoid, United Kingdom) or grown on Columbia agar (Difco, United States) containing 5% sheep blood (Ruite, China) in an incubator containing 5% CO_2_ at 37°C. *Escherichia coli* BL21 was cultured in Luria-Bertani (LB) medium in a shaking incubator at 37°C. The transformed strains were selected for growth in the presence of 4 μg/ml of chloramphenicol (Cm; Sigma, United States), or 100 μg/ml of ampicillin (Amp; Sigma, United States) were added to the medium.

The iron-restricted medium was prepared by adding 5% Chelex-100 resin (Bio-Rad, United States) to THY with continuous stirring for 8 h, then filtered to remove the resin, sterilized, and supplemented with 100 μM of CaCl_2_ and 2 mM of MgCl_2_. If necessary, an iron source, such as hemin or FeCl_3_, was added to the medium. All the bacterial strains and plasmids used in this study are listed in Table 1.

**TABLE 1:** Strains and plasmids used in this study.

Strain or plasmid	Relevant characteristics	Source
**Strains**		
***S. pneumoniae***		
D39(WT)	Wild type	ATCC (United States)
D39Δ*spd0090*	In-frame *spd0090* mutant strain derived from D39; Tet*^r^*	This study
D39Δ*spd0090+*	Δ*spd0090* strain transformed with pIB169-spd0090; Tet*^r^* Cm*^r^*	This study
D39Δ*spd0090169+*	Δ*spd0090* strain transformed with pIB169; Tet*^r^* Cm*^r^*	This study
D39Δ*piaA*Δ*piuA*Δ*pitA*	In-frame piuA/piaA/pitA mutant strain derived from D39; Erm*^r^* Cm*^r^* Spec*^r^*	Laboratory stock
***E. coil***		
BL21	Wild type	Invitrogen (United States)
BL21/pGEX-4T-1-*spd0090*	BL21 strain transformed with pGEX-4T-1*-spd0090*; Amp*^r^*	This study
**Plasmids**		
pGEX-4T-1	pGEX vector contained *tac* promoter; Amp*^r^*	Invitrogen (United States)
pGEX-4T-1-*spd0090*	*S. pneumoniae* D39 *spd0090* (33–492 AA) fragment cloned into pGEX-4T-1; Amp*^r^*	This study
plB169	Shuttle plasmid contained P *veg* promoter; Cm*^r^*	Laboratory stock
plB169-*spd0090*	*S. pneumoniae* D39 *spd0090* (1–492 AA) fragment cloned into pIB169; Cm*^r^*	This study

*Tet^r^, tetracycline resistance; Cm^r^, chloramphenicol resistance; Amp^r^, ampicillin resistance; AA, amino acids.*

### Construction of Mutant *S. pneumoniae* D39 Strains

D39Δ*spd0090* was constructed using the flanking homology polymerase chain reaction (LFH-PCR) (Wach, 1996) method, replacing the target gene *spd-0090* with the antibiotic resistance cassette gene (Tet, tetracycline, Sigma, United States). The LFH-PCR containing the upstream (705 bp) and downstream (652 bp) sequences of *spd-0090* and the Tet gene (1,501 bp) products were introduced into *S. pneumoniae D39* and incubated at 37°C for 2 h. The primers used for this work are listed in Table 2. The transformants were transferred to Columbia sheep blood agar plates containing Tet (3.5 μg/ml), and positive strains were selected in which the *spd*_*0090* gene had been completely replaced by the *tet* gene. D39Δ*spd0090* strain was stabilized after 7–8 consecutive passages in THY medium containing 3.5 μg/ml Tet. To construct the *spd-0090*-complement mutant strain, the *spd-0090* gene was inserted into the pIB169 plasmid using the ClonExpress II One Step Cloning Kit (Vazyme, China) method (Miao et al., 2018). The recombinant plasmid pIB169-*spd-0090* containing a Cm resistance cassette was transformed into strain D39Δ*spd0090*. The plasmid pIB169 was also transformed into strain D39Δ*spd0090* to construct as vector control. Transformants were screened with a Columbia sheep blood agar plate containing 4 μg/ml Cm. All mutant strains were further confirmed by Western blot.

**TABLE 2:** The primer sequences used in this study.

Primers	Sequence (5′–3′)
0090-P1	ATCCTACAAGCCCTCTTCATCTC
0090-P2	CCTACGATGGTTGGGTTG
0090-P3	ATCAAACAAATTTTGGGCCCGGAAGCACTATT CTTTCACACCACC
0090-P4	TCGTTAAGGGATCAACTTTGGGAAGTCGCTGA CGAGGGAAT
0090-F	GGTCTTCCATTCGTTATCG
0090-R	ATTTTTTCCCAGTTCTTGC
Tet-F	CCGGGCCCAAAATTTGTTTGAT
Tet-R	TCCCAAAGTTGATCCCTTAACGA
plB169-spd0090-F	CCGCGGTCCCGAATTCATGAAAAACTGGAAA AAATATGCTTTTG
plB169-spd0090-R	AGCGCTGAGACCATGGTTTATTTTTTGTTTTTCAA GAATTCATCGTATTG
pGE-X4T-1-spd0090-F	ACGCGTCGACTCGCTGATTCAGGTGACAAA
pGE-X4T-1-spd0090-R	ATAAGAATGCGGCCGCTTATTTTTTGTTTTTCAAG
16S rRNA-qPCR-F	CTGCGTTGTATTAGCTAGTTGGTG
16S rRNA-qPCR-R	TCCGTCCATTGCCGAAGATTC
*livJ-*qPCR-*F*	ACTTCCAAGCAGCCCTTACA
*livJ-*qPCR-*R*	ATCCATCACCACCAACGATT
*spd1609-*qPCR-*F*	TTCATCAAGGTAACGAACTAAGA
*spd1609-*qPCR-*R*	AAGAAATGCCTAAAGATTGGACT
*lytC*-qPCR-F	CATTCTCAGCATCGGTCTCA
*lytC*-qPCR-R	AGAACGAGGTGGATGGTGTC
*aliA*-qPCR-F	TGTGCTTGCGCCAGT
*aliA*-qPCR-R	ATTCAACAGAGGATTTGGTCA
*piuA*-qPCR-F	AGACTCACGCCACGGA
*piuA*-qPCR-R	TCTAGGACACCGTCGTTG

### Western Blot Analysis

PsaA, PiuA, and PiaA antibodies were derived from the previous work (Yang et al., 2016). The purified SPD_0090 protein was used as an antigen for the immunization experiments to generate multiclonal antibodies in mice according to the previous report (Brown et al., 2001b). Equal amounts of proteins were analyzed by 10–12% sodium dodecyl sulfate–polyacrylamide gel electrophoresis (SDS-PAGE) and then transferred to polyvinylidene fluoride (PVDF) membranes (Millipore, United States), which were incubated with the corresponding antibodies at 4°C. This was followed by incubation with horseradish peroxidase (HRP)-conjugated goat anti-mouse as a secondary antibody. Results were visualized with Clarity Western ECL substrate (Bio-Rad, United States) and captured using Image Master 2D Platinum 6.0 (GE Healthcare, United States).

### Real-Time Quantitative PCR

*S. pneumoniae* D39, D39Δ*spd0090*, and D39Δ*spd0090*^+^ strains at OD_600_ ∼ 0.6 in THY medium were collected by centrifugation and treated by 20 mg/ml lysozyme at 37°*C* for 30 min. The extraction of RNA and the synthesis of cDNA follow the previous methods (Dong et al., 2021).

Real-time quantitative PCR (RT-qPCR) was performed using the EvaGreen Dye (Bio-Rad, United States) with the TransStart Tip Green qPCR supermix kit (TransGen Biotech, China). The cycle threshold (CT) value was recorded, and the relative quantification of gene expression was calculated using the 2^–ΔΔ*CT*^ method, with 16S rRNA as an internal control. The results were compared to gene expression in the D39Δ*spd0090* or D39Δ*spd0090*^+^ with gene expression in the D39 strain. All data were obtained from three independent biological experiments. The primers used for RT-qPCR are shown in Table 2.

### Cloning, Expression, and Purification of SPD_0090

The *spd_0090* gene, encoding the SPD_0090 protein without a signal peptide, was cloned from *S. pneumoniae* D39 by PCR, and the pGEX-4T-1 vector was digested with *Sal*I and *Not*I (TaKaRa, Japan) and ligated with T4 DNA ligase (TaKaRa, Japan). The fused vector pGEX-4T-1-*spd-0090* was confirmed by DNA sequencing (Tsingke, China) and then transformed to *Escherichia coli* BL21 followed by culturing in an LB medium containing 100 μg/ml Amp at 37°C in a shaking incubator. The expression and purification of SPD_0090 protein were performed according to the previous method (Zheng et al., 2021).

### Extraction of Membrane Protein

*S. pneumoniae* D39 was cultured in THY to OD_600_ ∼ 0.6, collected by centrifugation, and washed three times with phosphate-buffered saline (PBS). The collected cells were resuspended in 800 μl PBS with 1 mM phenylmethylsulfonyl fluoride (PMSF) and 0.5 mg/ml lysozyme, digested at 37°C for 30 min, then centrifuged at 3,000 × *g* for 5 min, and the pellet was discarded. A final concentration of 2% Triton X-114 was added to the supernatant and incubated at 4°C for 1 h (Bordier, 1981; Khandavilli et al., 2008). The sample was then transferred to a water bath at 37°C and incubated for 10 min to induce phase separation. The membrane proteins were separated by centrifugation at 10,000 × *g* for 10 min at room temperature, and the upper aqueous phase and the lower membrane phase were used separately for SDS-PAGE.

### UV/Vis Spectrum

The purified SPD_0090 (50 μM) was incubated with an excess of hemin (200 μM) for 2 h at room temperature and passed through a desalination column and a 10-kDa ultrafiltration column (Millipore, United States) (Cao et al., 2018). Then, the sample was washed six times with 20 mM Tris-HCl (pH 7.4, containing 100 mM NaCl) in a 10-kDa ultrafiltration column by centrifugation to remove the hemin unbound to SPD_0090, and the remaining sample was the hemin-saturated SPD_0090 protein, named SPD_0090 + hemin. The hemin solutions were prepared in 20 mM Tris-HCl containing 100 mM NaCl (pH 7.4) at a concentration of 25 μM, and 500 μl of SPD_0090 and SPD_0090 + hemin solutions were scanned from 200 to 600 nm by UV spectrophotometer (Evolution 300, Thermo Fisher Scientific, United States), and each experiment was repeated three times.

### Fluorescence Spectrometry

The binding of SPD_0090 to hemin was detected by a fluorescence spectrometer (F7000, Hitachi, Japan). A concentration of 2 μM SPD_0090 was prepared with 20 mM Tris-HCl (pH 7.4, containing 100 mM NaCl) buffer, and a high concentration of 1.2 mM hemin solution was prepared with the same buffer. The parameter settings are the same as earlier (Miao et al., 2018). The fluorescence spectra of SPD_0090 from 300 to 450 nm were recorded after that 1.2 mM hemin was gradually added to 1.5 ml of 2 μM SPD_0090 until the fluorescence intensity stabilized. Each scan was repeated three times and the titration curves were analyzed in Origin version 9.0, and the binding affinity (Ka) was calculated using Hill plots.

### Electron Paramagnetic Resonance Spectroscopy

A buffer of 20 mM Tris-HCl (pH 7.4, containing 100 mM NaCl) was used to prepare 400 μl each of 100 μM hemin and SPD_0090 + hemin solution (SPD_0090: hemin = 1:1). The electron paramagnetic resonance spectra of hemin and SPD_0090 + hemin solution were measured by a Bruker spectrometer (Elexsys E580, Bruker, Germany) with the following parameters: frequency, 9.4 GHz; power, 4 mW; modulation amplitude, 3 G; modulation frequency, 100 kHz; and temperature, 10 K. The obtained data were processed and plotted using the Origin version 9.0 software.

### Cell Culture, Adherence, and Invasion Assays

The A549 human alveolar epithelial cell line (ATCC: CCL-185) was cultured in Dulbecco’s modified eagle medium (DMEM) (Life Tech Technologies, United States), supplemented with 10% fetal bovine serum (FBS, Gibco, United States), and incubated at 37°C in 5% CO_2_. Cells were transferred to 12-well plates and cultured to uniformly full cell layers (2 × 10^5^ cells/well) for adhesion and invasion assays. Bacterial adhesion and invasion assays were performed essentially, as previously described, with minor modifications (Quin et al., 2007; Yang et al., 2019). Briefly, D39 and D39Δ*spd0090* strains were cultured to OD_600_ ∼ 0.6 and then diluted to 1 × 10^7^ CFU/ml. The collected bacteria were resuspended in a DMEM containing 1% FBS and added to cell plates and incubated in a CO_2_ incubator for 2 h.

For the adhesion assay, A549 cells were washed three times with sterile PBS to wash away unadhered bacteria. Cells were lysed by adding 1% Triton X-100 for 30 min on ice, diluted in a gradient, and spread on Columbia agar plates containing 5% sheep blood.

For the invasion assay, DMEM contained 100 μg/ml of gentamicin (Gen, Sigma, United States) and 1% FBS was added and incubated for 30 min at 37°C, followed by three washes with sterile PBS. Cells were lysed by adding 1% Triton X-100 for 30 min on ice, diluted from 10^1^ to 10^5^ times in a gradient, and spread on Columbia agar plates containing 5% sheep blood. Three independent biological experiments were repeated.

### Animal Experiments

All animals used in the experiments were purchased from the Experimental Animal Management Center of Southern Medical University (Guangdong, China), Female BALB/c mice between 6 and 8 weeks of age were used in our experiments. For animal infection experiments, the D39 and D39Δ*spd0090* strains were grown in THY to logarithmic growth phase, collected, and washed three times with PBS. The bacterial solution (2.5 × 10^8^ CFU/20 μl) was dropped into the nose of the mice. The infected mice were observed for survival transitions; nasal lavage and lung tissue were collected at 12 or 24 h. The number of viable bacteria was determined by serial dilutions applied to a Columbia medium containing 5% sheep blood. Survival times were calculated using the GraphPad Prism software and analyzed by the log-rank (Mantel–Cox) test (Robb et al., 2017). The number of bacteria in the nasal lavage solution and lungs was analyzed by the Mann–Whitney test. A value of *p* < 0.05 was considered statistically significant.

### Growth Curve Analysis

The growth curves of the bacteria in restriction medium C + Y were determined by UV-visible spectroscopy (Evolution 300, Thermo Fisher Scientific, United States) (Miao et al., 2018). The medium was configured at room temperature and filtered through a sterile 0.22-μm filter membrane to remove bacteria. *S. pneumoniae* D39, D39Δ*spd0090*, D39Δ*spd0090*^169+^, and D39Δ*spd0090*^+^ strains were inoculated into the C + Y medium (no sugar), with galactose (10 mM), glucose (8 mM), and lactose (8 mM) at equivalent inoculation doses at 37°C in 5% CO_2_ incubator. *S. pneumoniae* D39, D39Δ*spd0090*, D39Δ*spd0090*^+^, and D39Δ*spd0090*^169+^ strains were inoculated into normal THY medium, iron-restricted medium containing iron chelator 2,2′-bipyridine (1 mM), or iron-restricted culture supplemented with 25 μM of hemin. The iron-restricted THY medium was prepared by adding 1 mM 2,2-bipyridine (DP) (Sigma-Aldrich), which was filtered with a 0.22-μM filter to THY, and incubated at 37°C for 2 h. The optical density at 600 nm (OD_600_) was continuously measured every 2 h by UV-visible spectroscopy) for 12 h. All data were analyzed by using GraphPad Prism version 8.0.2 (GraphPad Software, United States).

### Protein Preparation, iTRAQ Labeling, and Proteomics Analysis

Total proteins were extracted from D39 and D39Δ*spd0090* in the exponential growth phase when they were cultured in the THY medium. A volume of 500 μg of total protein was taken from each sample and digested with trypsin (Promega, United States) at 37°C for 18 h and lyophilized. Then, an iTRAQ Reagent multiplex kit (AB Sciex, United States) was used to label peptide samples according to the manufacturer’s protocol. The two groups of samples were labeled separately at room temperature for 1 h and then the labeled samples were mixed in equal proportions and lyophilized (Group 1: “115” labels D39, “113” labels D39Δ*spd0090*; Group 2: “119” labels D39, “113” labels D39Δ*spd0090*).

The labeled samples were grouped using high-performance liquid chromatography (HPLC; WuFeng, China), referring to the previous description for the specific experimental steps (Zheng et al., 2021). Then, the samples were desalted according to the instructions for the MonoTip C18 (SHIMADZU, Japan) desalting column. Desalted peptides were resuspended with buffer A (5% acetonitrile, 0.1% formic acid) and detected using an Orbitrap Fusion Lumos mass spectrometer (Thermo, United States). Then, the raw data obtained were identified and quantified by using the Proteome Discoverer software version 2.1 (Thermo Fisher Scientific, United States) and searched using the *S. pneumoniae* D39 database. The specific parameters were as follows: Sample Type, iTRAQ 8plex (peptide labeled); Digestion, Trypsin; instrument, Orbitrap Fusion Lumos; Variable modifications, Oxidation (M), Acetyl (Protein N-term); search effort, thorough; and detected protein threshold [unused ProtScore (Conf)] > 1.30 (95.0%). In this study, the cutoff threshold for increased and decreased abundance of the differentially expressed proteins was defined using the population statistics applied to the biological replicates reported by Gan et al. (2007). The finalized cutoff values for increased (1.4) and decreased (0.71) protein abundance were determined as the threshold. Gene Ontology (GO) and Kyoto Encyclopedia of Genes and Genomes (KEGG) enrichment analysis were performed in Glue GO plug-in of Cytoscape (version 3.9.0) with *p* < 0.05 to analyze the biological processes of differentially expressed proteins (Shannon et al., 2003).

### Prediction of Protein Location

According to the protein sequence of SPD_0090 as indicated by NCBI^1^, the analysis of SPD_0090 localization by using PSORTb version 3.0.3^2^ (Yu et al., 2010) and the analysis of SPD_0090 signal peptide prediction by using SignalP - 5.0^3^ (Armenteros et al., 2019) were performed.

### GST Pull-Down Assay

The expression and purification of GST_SPD_0090 and Glutathione-S-transferase (GST) protein were carried out by the above-mentioned method (Zheng et al., 2021). GST_SPD_0090 solubilized to glutathione-Sepharose (GE Healthcare, United States) beads was incubated with D39 cell lysate in PBS buffer overnight at 4°C with the GST as the negative control. Beads were washed with IP Lysate buffer (Beyotime, China) to remove the unbound proteins. Proteins bound to the beads were collected in PBS buffer by centrifugation and heated at 100°C. The isolated proteins were separated by SDS-PAGE, stained by AgNO_3_, then digested by trypsin, and identified by mass spectrometry (MS) (Zhou et al., 2021) (Orbitrap Fusion Lumos; Thermo Fisher Scientific).

### Statistical Analysis

Data were statistically analyzed by using GraphPad Prism version 8.0.2. The differences between the two groups were analyzed by two-tailed, unpaired Student’s *t*-tests, and data are expressed as mean ± standard (SD). Results were considered significant at *p* ≤ 0.05 (denoted by *), *p* ≤ 0.01 (denoted by ^**^), *p* ≤ 0.001 (denoted by ^***^), and *p* ≤ 0.0001 (denoted by ^****^). The data on the survival of mice for the virulence experiment were analyzed using the log-rank (Mantel–Cox) test, and a number of CFUs in the different experimental groups were compared using the Mann–Whitney test.

### Data Availability

The MS proteomics data have been deposited to the ProteomeXchange Consortium *via* the PRIDE (Deutsch et al., 2020) partner repository with the dataset identifier PXD030183^4^ (username: reviewer_pxd030183@ebi.ac.uk; password, 5LSbJZVk).

## Results

### SPD_0090 Is Highly Conserved in Gram-Positive Bacteria

**FIGURE 1:**
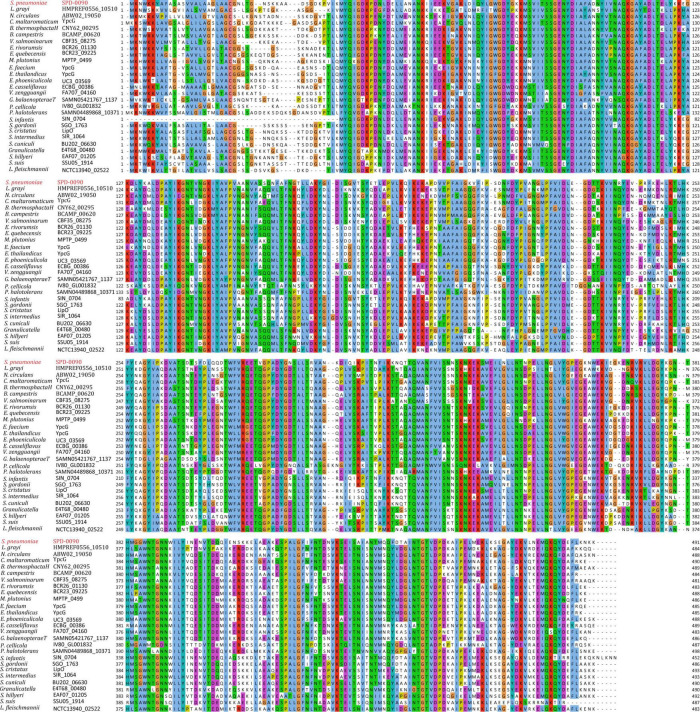
The multiple sequence alignment of SPD_0090 with homologous proteins in various bacterial species.

To assess the conservation of SPD_0090 in bacteria, amino acid sequence alignment was carried out in this study (Figure 1). The result showed that the SPD_0090 homologous proteins are highly conserved in Gram-positive bacteria. Several highly homologous proteins of SPD_0090, e.g., SGO_1763, BCR26_01130, and CBF35_08275, are annotated as sugar ABC transporter substrate-binding proteins in the Uniprot database, but no experimental data studies reveal their function so far. As mentioned in the introduction section, our previous study has suggested that SPD_0090 may be associated with iron transportation in *S. pneumoniae* (Yang et al., 2016). In this study, we used a biochemical method combined with quantitative proteomics to investigate the potential biological function of SPD_0090 in *S. pneumoniae.*

### The Deletion of the *spd*_*0090* Gene Hinders the Growth of *S. pneumoniae*

**FIGURE 2:**
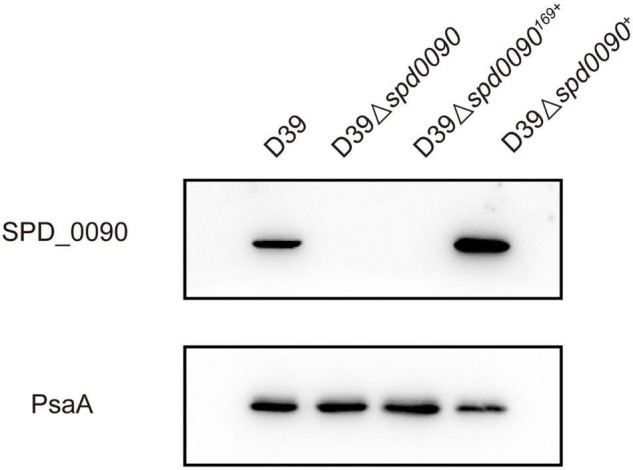
The construction and verification of the D39Δ*spd0090*, D39Δ*spd0090*^169+^, and D39Δ*spd0090*^+^ strains. Whole-cell proteins isolated from D39, D39Δ*spd0090*, D39Δ*spd0090*^169+^, and D39Δ*spd0090*^+^ were extracted to perform Western blot to validate gene knockout and complement strains. PsaA was used as loading controls.

To find out the detailed biological functions of SPD_0090 in *S. pneumoniae*, the D39Δ*spd0090* strain was first constructed by homologous substitution, and the complemented mutant D39Δ*spd0090*^+^ was obtained by supplementing the plasmid pIB169-*spd*_*0090* into the D39Δ*spd0090* strain; pIB169 was introduced to the D39Δ*spd0090* strain to obtain D39Δ*spd0090*^169+^. Western blot showed that the SPD_0090 protein was highly expressed in D39Δ*spd0090*^+^ (Figure 2), while it was undetectable in D39Δ*spd0090* and D39Δ*spd0090*^169+^. These results indicated the successful construction of the gene knockout and complement strains.

**FIGURE 3:**
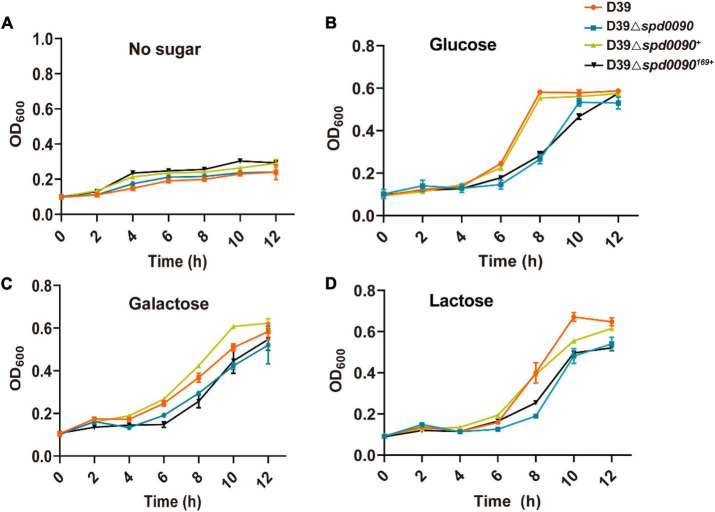
The deletion of *spd*_*0090* gene affects the growth of *S. pneumoniae.* **(A–D)** Growth curves of D39 (red), D39Δ*spd0090* (blue), D39Δ*spd0090*^169+^ (black), and D39Δ*spd0090*^+^ (yellow) strains cultured in C + Y medium containing no sugar, glucose galactose, and lactose. *N* = 3, each group of experiments was repeated three times.

We first checked whether SPD_0090 affects the growth of *S. pneumoniae*. The growth curves showed that D39, D39Δ*spd0090*, D39Δ*spd0090*^169+^, and D39Δ*spd0090*^+^ strains barely grew in the C + Y medium without any sugar source (Figure 3A). However, previous studies have speculated that SPD_0090 is a galactose ABC transporter protein (Durmort and Brown, 2015), in the presence of glucose, galactose, and lactose; the growth rate of D39Δ*spd0090* and D39Δ*spd0090*^169+^ was lower than that D39 (Figures 3B–D), but the growth rate of D39Δ*spd0090*^+^ growth was close to that of D39. We counted the data of D39 and D39Δ*spd0090* in the logarithmic phase of growth and found that the growth of D39Δ*spd0090* is significantly slower than that of D39 cultured in three sugar sources even at a high concentration of sugars (Supplementary Figure 1). The above results suggest that the knockout of the *spd_0090* gene resulted in a restricted growth of *S. pneumoniae*.

### SPD_0090 Affects the Ability of *S. pneumoniae* to Use Hemin

**FIGURE 4:**
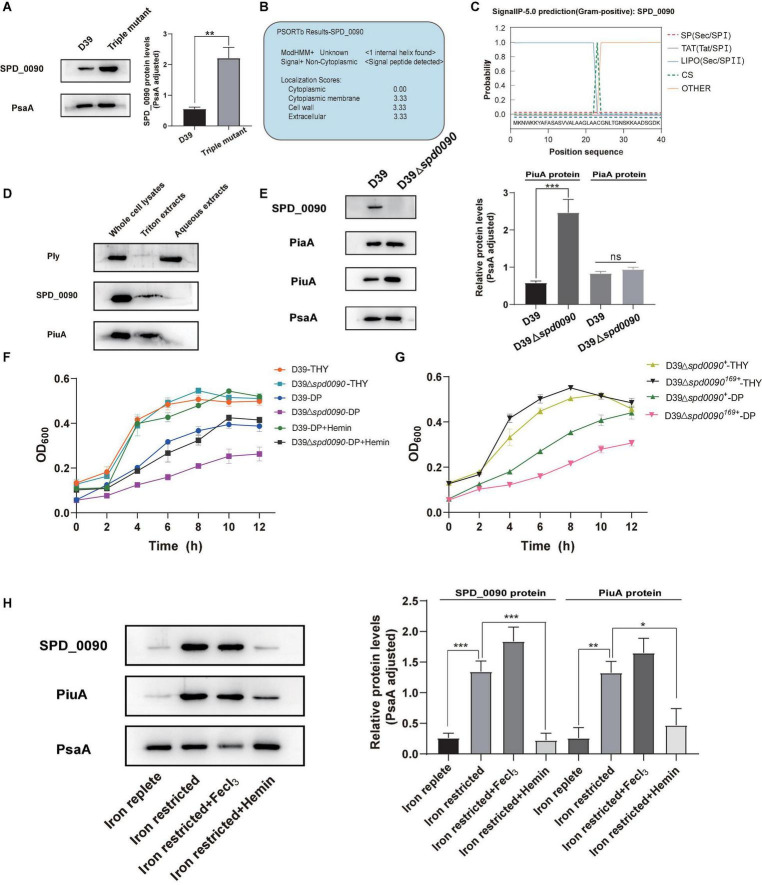
The deletion of the *spd*_*0090* gene affects the ability of *S. pneumoniae* to use hemin. **(A)** Western blot analysis of the protein levels of SPD_0090 in D39 and Triple Mutant strains (D39*ΔpiaAΔpiuAΔpitA*). **(B)** The PSORTb analysis of SPD_0090 protein. **(C)** The SignaI P 5.0 analysis of SPD_0090 protein. **(D)** Western blot analysis of SPD_0090 as a membrane protein. The protein of SPD_0090, PiuA, and Ply in whole cell lysates, Triton X-114 isolated membrane protein phase and aqueous phase was determined by Western blot. **(E)** The protein levels of PiaA and PiuA in THY-extracted D39 and D39Δ*spd0090* whole-cell lysates determined by Western blot. **(F)** Growth curves of the D39 and D39Δ*spd0090* strains in iron-replete, iron-restricted, and iron-restricted supplement 25-μM hemin. Data are presented as the mean ± SEM from three independent growth curves. **(G)** Growth curves of the D39Δ*spd0090*^169+^ and D39Δ*spd0090*^+^ strains under iron-replete (THY) and iron-restricted conditions (1 mM 2, 2′-dipyridyl, DP). **(H)** SPD_0090 and PiuA protein levels in whole proteins extracted from D39 cultured in iron-replete, iron-restricted, iron-restricted + FeCl_3_, and iron-restricted + hemin media. PiuA served as a positive control. The figure of Western blot is representative of at least three independent replicates. Western blot data were quantified using ImageJ, normalized by PsaA and analyzed by GraphPad Prism version 8.0.2. Data were analyzed by two-tailed, unpaired Student’s *t*-tests, and results are expressed as means ± SD. ***p < 0.001, **p < 0.01, *p < 0.05. ns, no significance.

To study the relationship between SPD_0090 and iron metabolism in *S. pneumoniae*, we detected the SPD_0090 protein level in D39 and the triple mutant (Δ*piuA/*Δ*piaA/*Δ*pitA*) in which three genes encoding major iron transporters were simultaneously knocked out. There were significantly higher levels of SPD_0090 protein in the triple mutant, relative to D39 (Figure 4A), which is consistent with the previous iTRAQ-based quantitative proteomics results of our lab (Yang et al., 2016). Furthermore, PSORTb prediction showed that SPD_0090 is not an intracellular protein (Figure 4B). Then, we used signal prediction and found that SPD_0090 is a lipoprotein anchored in the cell membrane, which contains a cleavage site of typical lipoprotein signal peptide (between positions 23 and 24 amino acids of the SPD_0090 protein sequence) (Figure 4C). We isolated membrane proteins from *S. pneumoniae* D39 by using Triton X-114 and carried out a Western blot analysis (Bordier, 1981). A well-known membrane protein PiuA was used as a positive control, and the soluble protein Ply was present in the aqueous phase as a negative control. As shown in Figure 4D, SPD_0090 is present in the whole cell lysate and membrane protein fractions, but not in the upper aqueous phase, demonstrating that SPD_0090 is a membrane protein.

To further study which form of iron is associated with SPD_0090, two major proteins of PiuA (hemin transporter) and PiaA (ferrichrome transporter) were detected in D39 and D39Δ*spd0090* cultured in the THY medium by Western blot. There was a higher level of PiuA in D39Δ*spd0090*, relative to D39, while there was no significant difference in PiaA protein levels between the two strains (Figure 4E). As PiuA is responsible for hemin uptake on the cell surface, we speculated that SPD_0090 may be involved in hemin transportation. We detected the growth curves of D39, D39Δ*spd0090*, D39Δ*spd0090*^169+^, and D39Δ*spd0090*^+^ under iron-abundant (THY) and iron-restricted conditions (1 mM 2,2′-bipyridine, DP). As shown in Figures 4F,G in the THY medium, there was almost no difference in the growth of D39 and D39Δ*spd0090*, D39Δ*spd0090*^169+^, and D39Δ*spd0090*^+^. In the condition of the iron restriction, the growth of D39Δ*spd0090* was significantly lower than that of D39 (Figure 4F). The growth of D39Δ*spd0090*^169+^ was significantly lower than that of D39Δ*spd0090*^+^ (Figure 4G). When hemin was supplemented in the iron-restricted medium of D39 and D39Δ*spd0090*, the growth of D39 returned close to that in the THY medium when the growth of D39Δ*spd0090* was only partially recovered (Figure 4F). This result indicated that the deletion of *spd_0090* may affect the ability of *S. pneumoniae* to use hemin and thus result in diminished growth. Then, we examined whether the SPD_0090 protein is regulated by hemin in the medium. As shown in Figure 4H, SPD_0090 protein had higher levels in iron-restricted culture conditions relative to iron-replete conditions, suggesting that SPD_0090 is evidently regulated by iron concentration. Then, we supplemented hemin and FeCl_3_ as an iron source to the iron-restricted medium. The Western blot analysis showed that the level of SPD_0090 protein was decreased to a quarter of the original level (in the iron-restricted medium) after the supplement of 25 μM hemin, while it remained almost unchanged upon 25 μM FeCl_3_ addition (Figure 4H). These changes in SPD_0090 abundance in response to hemin are similar to those of PiuA (Figure 4H). Therefore, based on the above results, we hypothesized that SPD_0090 is a potential hemin transporter located on the cell surface.

**FIGURE 5:**
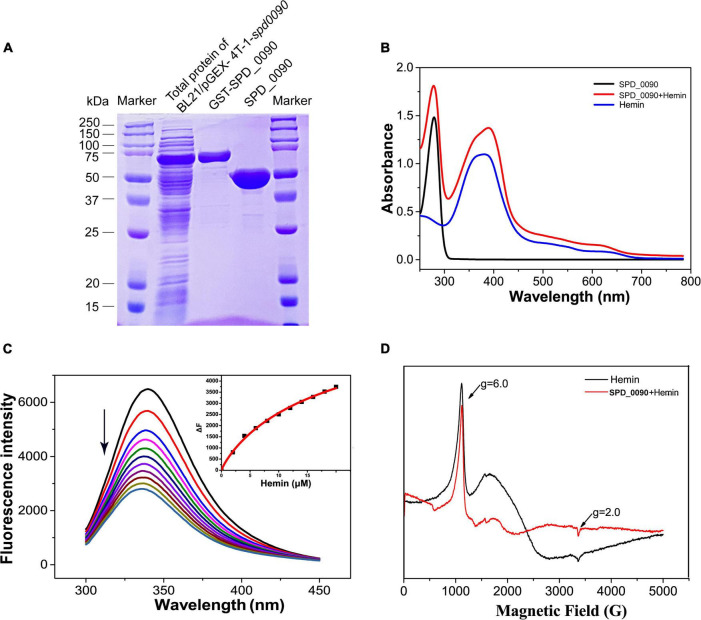
SPD_0090 binds to hemin *in vitro*. **(A)** SPD_0090 was expressed with pGEX-4T-1-GST fusion system and purified by Glutathione Sepharose. **(B)** UV/vis spectra of the SPD_0090, Hemin, SPD_0090 + Hemin. The protein concentration is 25 μM and the spectra were scanned from 200 to 600 nm. **(C)** Fluorescence spectra of hemin titration to SPD_0090 (2 μM). **(D)** EPR spectrum of SPD_0090 in combination with hemin and hemin alone. All experiments were repeated three times. Statistical analysis was conducted using Origin version 9.0.

Next, we expressed, purified SPD_0090 protein, and *in vitro* characterized its hemin binding properties (Figure 5A). In the UV/vis spectra of Hemin, a broad absorption peak at around 385 nm was observed which is the typical absorption peak of hemin. In the UV spectra of SPD_0090-hemin, the characteristic absorption peak of the band of hemin was shifted from 385 to 410 nm which is a typical spectrum of hemin-binding protein (Figure 5B). Subsequent fluorescence spectroscopy detection showed that hemin binding to SPD_0090 resulted in fluorescence quenching at 340 nm. The fluorescence titration curve was fitted to a Hill plot, and the binding constant (Ka) of SPD_0090 to hemin was calculated to be 4.6 × 10^4^M^–1^ (Figure 5C). Then, we further used EPR to investigate the nature of hemin binding to SPD_0090. As shown in Figure 5D, the single peak signal in the high-spin (HS) region [HS, 900–1,900 G] at g = 6.0 and the low-spin (LS) region (LS, 2,300–4,300 G) at g = 2.0 is a characteristic peak of hemin (Carette et al., 2006; Lu et al., 2010). After binding with SPD_0090, the EPR feature of hemin was changed at the peaks (Figure 5D). The above results suggested that SPD_0090 can bind hemin *in vitro*.

### SPD_0090 Negatively Contributed to Adherence and Invasion of A549 Cells

**FIGURE 6:**
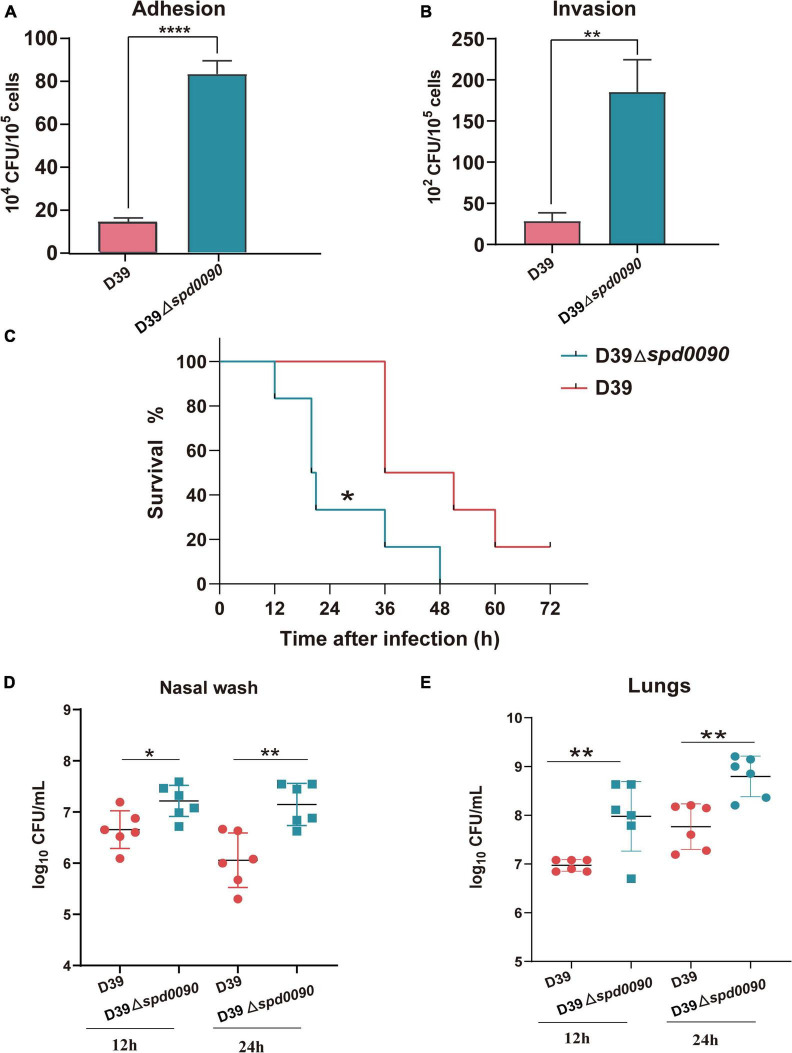
SPD_0090 negatively regulates the virulence of *S. pneumoniae.* **(A,B)** The adherence and intracellular invasion ability of D39 and D39Δ*spd0090* strains. **(C)** The survival curves of D39 or D39Δ*spd0090* strains infected mice *via* nasal infection with 2.5 × 10^8^ CFU/20 μl. *n* = 6 animals per group. Survival was analyzed using the log-rank (Mantel–Cox) test between the D39 and D39Δ*spd0090* strains. **(D,E)** Bacterial burdens of the D39 and D39Δ*spd0090* strains in the nasal lavage solution and lungs of mice 12 or 24 h post-challenge. Horizontal lines represent median values. *p* < 0.05 for comparison by the Mann–Whitney test to the results for mice infected with the parental WT; *n* = 6 animals per group. Error bars represent the SEM. Data (adherence and intracellular) were analyzed by two-tailed, unpaired Student’s *t*-tests, and results are expressed as means ± the SD (* indicates *p* < 0.05, ** indicates *p* < 0.01, *** indicates *p* < 0.001, and **** indicates *p* < 0.0001).

Some iron transporters of pathogenic bacteria also directly or indirectly affect adhesion and invasion of the host cells (Durmort and Brown, 2015; Nguyen and Götz, 2016). Therefore, we investigated whether SPD_0090 is also important to *S. pneumoniae* infection in human lung cancer cells (A549). The adhesion and invasion ability of D39 and D39Δ*spd0090* to A549 cells were measured. As shown in Figures 6A,B, compared to D39, D39Δ*spd0090* showed a fourfold and a fivefold increase in the number of adherent and invasive bacteria, respectively (Figures 6A,B). This result indicated that SPD_0090 negatively contributes to *S. pneumoniae* infection of human lung carcinoma cells.

### The Animal Model Reveals the Importance of SPD_0090 for the Infection Ability of *S. pneumoniae*

To fully dissect the effect of SPD_0090 on the ability of *S. pneumoniae* to infect the host, we used intranasally infected mice (pneumonia model) to assess bacterial virulence. After infection with D39, mice began dying after 36 h, while their survival rate at 72 h remained at 16%. The infection of the knockout strain was completely different from that of the WT. After infection with D39Δ*spd0090*, mice began to die by 12 h and all died at 48 h. This result indicated that the mortality rate of infection with D39Δ*spd0090* is significantly higher than that with D39 (Figure 6C). To further test the ability of D39 and D39Δ*spd0090* strains to invade host tissue cells, we collected nasal lavage and lungs from mice at 12 and 24 h post-infection and plated them on the agar, followed by CFU counting. At both time points, bacterial counts of D39Δ*spd0090* in nasal and lung were higher than that of D39 (Figures 6D,E). This invasion resulting in the animal model is consistent with the conclusion derived from the infection of A549 cells. The combined results emphasized that SPD_0090 protein negatively regulates the virulence of *S. pneumoniae* which is different from the most known iron transporters.

### iTRAQ-Based Proteomics Revealed the Effect of SPD_0090 on Biological Pathways of *S. pneumoniae*

**FIGURE 7:**
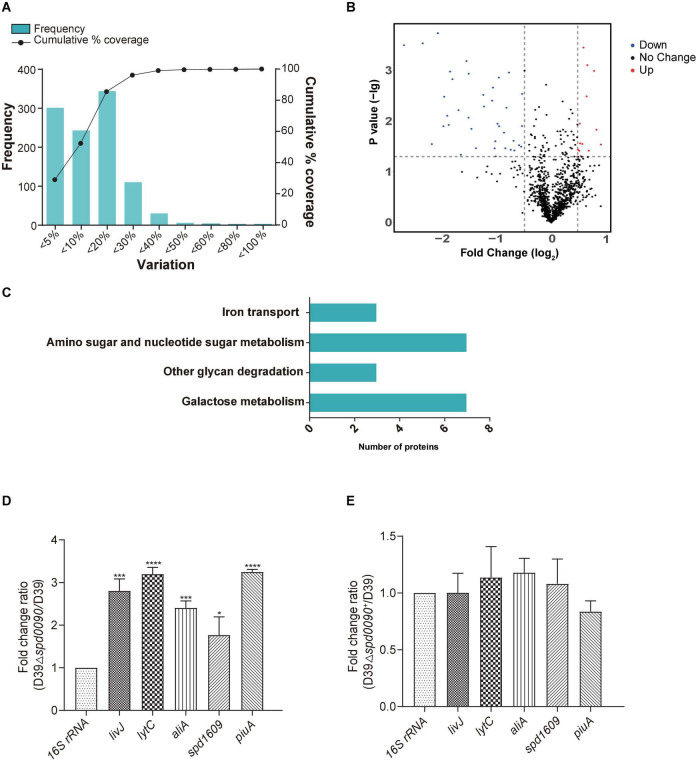
The results of iTRAQ-based proteomics quantitative analysis. **(A)** Distribution of biological variables in two replicates. **(B)** The volcano map of the differentially expressed proteins. Each dot represents the average level of individual proteins obtained from two independent biological experiments. The red and blue dots were recognized as differentially expressed proteins according to the fold change, which was more than 1.4-fold or less than 0.71-fold, and *p* < 0.05. **(C)** Enriched pathway for differentially expressed proteins. **(D,E)** The relative mRNA expression levels of genes involved in virulence regulation were determined by RT-qPCR. All results represent the relative expression levels of strain D39Δ*spd0090* vs. D39 strain or D39Δ*spd0090*^+^ strain vs. D39 strain. The results as the mean value (±SEM) from three independent biological experiments. Data were compared to 16S rRNA as determined by the Student’s *t*-test analysis (* indicates *p* < 0.05, ** indicates *p* < 0.01, *** indicates *p* < 0.001 and **** indicates *p* < 0.0001).

To investigate the mechanism of the SPD_0090 effect on the virulence of *S. pneumoniae*, we performed iTRAQ-based proteomics to find out the biological processes changed by the *spd-0090* gene knockout. In total, 1,037 proteins were identified in D39 and D39Δ*spd0090*. The threshold for defining differential expressed proteins in this experiment was defined using the biological replicate method reported by Gan et al. (2007). The analysis of protein coefficient of variation was performed for the proteins identified in this experiment, and 88% of the proteins had coefficients of variation ≤ 40% (Figure 7A), so the biological variation threshold for this experiment was set at 40%. The threshold for fold change of differentially expressed proteins was defined as ≥1.4 for increased abundance or ≤0.71 for decreased abundance with *p* < 0.05 (Figure 7B). In the two biological replicates, there were 52 differentially expressed proteins in D39Δ*spd0090* compared to D39 (Table 3). The GO analysis indicated that 52 differentially expressed proteins were enriched in several metabolic pathways, including galactose metabolism, amino sugar and nucleotide sugar metabolism, iron transport, and other glycan degradations (Figure 7C).

**TABLE 3:** Differentially expressed proteins identified in D39Δ*spd0090*.

Gene No.	Protein name	Gene name	Fold	*p*-value
**Carbohydrate metabolic process**				
SPD_0562	Beta-galactosidase, putative	*bgaA*	0.50	0.011
SPD_0065	Beta-galactosidase 3	*bgaC*	0.34	0.001
SPD_1634	Galactokinase	*galK*	0.35	0.001
SPD_0071	Aldose 1-epimerase	*galM*	0.27	0.001
SPD_1633	Galactose-1-phosphate uridylyltransferase	*galT*	0.35	0.008
SPD_1052	Galactose-6-phosphate isomerase subunit	*lacB*	0.38	0.034
SPD_1050	Tagatose 1,6-diphosphate aldolase	*lacD*	0.49	0.049
SPD_1866	*N*-acetylglucosamine-6-phosphate deacetylase	*nagA*	0.47	0.003
SPD_0063	Beta-*N*-acetylhexosaminidase	*strH*	0.36	0.014
SPD_0070	Sugar isomerase domain protein	*agaS*	0.19	0.001
SPD_1996	Fucose operon repressor, putative	*spd*_*1996*	0.66	0.029
SPD_0301	Sugar binding transcriptional regulator	*regR*	0.71	0.001
**Carbohydrate transport**				
SPD_0066	PTS system, IIB component	*spd*_*0066*	0.28	0.001
SPD_0067	PTS system, IIC component	*spd*_*0067*	0.26	0.007
SPD_0068	PTS system, IID component	*spd*_*0068*	0.23	0.001
SPD_0561	PTS system, IIC component, putative	*spd*_*0561*	0.47	0.002
SPD_0560	PTS system, IIB component, putative	*spd*_*0560*	0.48	0.034
**ABC transport**				
SPD_0150	ABC transporter, substrate-binding protein	*spd*_*0150*	1.44	0.027
SPD_1328	Amino acid ABC transporter, amino acid-binding protein	*aatB*	1.46	0.049
SPD_1137	ABC transporter, ATP-binding protein	*spd*_*1137*	1.57	0.003
SPD_1652	Iron-compound ABC transporter, iron-compound-binding protein	*spd*_*1652*	1.58	0.001
SPD_0334	Oligopeptide ABC transporter, oligopeptide-binding protein	*aliA*	1.49	0.028
SPD_0652	Branched-chain amino acid ABC transporter, amino acid-binding protein	*livJ*	1.61	0.038
SPD_0719	Amino acid ABC transporter, permease protein OS = Streptococcus pneumoniae	*spd*_*0719*	1.78	0.014
SPD_1609	ABC transporter, substrate-binding protein	*spd*_*1609*	1.88	0.028
SPD_0090	ABC transporter, substrate-binding protein	*spd*_*0090*	0.32	0.045
SPD_0614	ABC transporter, ATP-binding protein	*spd*_*0614*	0.42	0.003
**Other**				
SPD_1231	Phosphate transport system permease protein	*spd*_*1231*	1.41	0.036
SPD_0752	Membrane protein, putative	*spd*_*0752*	1.43	0.038
SPD_0582	Uncharacterized protein	*spd*_*0582*	1.44	0.011
SPD_1403	1,4-beta-*N*-acetylmuramidase, putative	*lytC*	1.51	0.001
SPD_1258	Peptidase, U32 family protein	*spd*_*1258*	1.73	0.001
SPD_0091	UPF0176 protein SPD_0091 [tRNA uridine(34) hydroxylase]	*spd*_*0091*	0.15	0.001
SPD_1588	Uncharacterized protein	*spd*_*1588*	0.22	0.028
SPD_1301	NADPH-dependent FMN reductase	*spd*_*1301*	0.25	0.012
SPD_1590	General stress protein 24, putative	*spd*_*1590*	0.25	0.003
SPD_0610	Uncharacterized protein	*spd*_*0610*	0.27	0.011
SPD_0335	Cell wall surface anchor family protein	*spd*_*0335*	0.29	0.001
SPD_1591	Uncharacterized protein	*spd*_*1591*	0.31	0.006
SPD_0816	Cof family protein	*spd*_*0816*	0.49	0.024
SPD_1365	Uncharacterized protein	*spd*_*1365*	0.51	0.001
SPD_1865	Alcohol dehydrogenase, zinc-containing	*spd*_*1865*	0.51	0.012
SPD_1118	Copper homeostasis protein	*cutC*	0.55	0.016
SPD_0525	Sensor histidine kinase	*vncS*	0.56	0.034
SPD_1903	DNA mismatch repair protein	*mutS*	0.56	0.005
SPD_0524	DNA-binding response regulator	*vncR*	0.58	0.001
SPD_0786	Arginine repressor	*argR*	0.60	0.031
SPD_0024	Adenylosuccinate synthetase	*purA*	0.62	0.024
SPD_0700	Aminopeptidase *N* (metallopeptidase activity zinc ion binding)	*pepN*	0.62	0.037
SPD_1927	Cation-transporting ATPase, E1-E2 family protein	*spd -1927*	0.69	0.012
SPD_0320	2T	*cps2T*	0.69	0.002
SPD_0126	Pneumococcal surface protein A	*pspA*	0.42	0.005

*Fold represents fold changes of proteins in D39Δspd0090 vs. D39.*

During the colonization and infection process, *S. pneumoniae* must obtain a sugar source from the host environment to support their survival (Palmer and Skaar, 2016; Lopez and Skaar, 2018). The quantitative proteomics data showed that many proteins that participated in carbon metabolism and sugar transportation had a lower abundance in D39Δ*spd0090* compared to that in D39. For example, galactokinase (GalK), aldose-1-epimerase (GalM), galactose-1-phosphate (GalT), galactose-6-phosphate isomerase subunit (LacB), tagatose 1,6-diphosphate aldolase (LacD), and galactose transport [three components of phosphotransferase (PTS) system] (Table 3). This result indicated that the deletion of the *spd_0090* gene reduced the expression of catabolic pathways responsible for energy production which is why the knockout stain grew slow.

Notably, 1,4-beta-*N*-acetylmuramidase (LytC), oligopeptide-binding protein (AliA), and amino acid-binding protein (LivJ) were present at relatively higher levels in D39Δ*spd0090* and known to be involved in nasopharyngeal epithelial colonization as well as *S. pneumoniae* invasion (Kerr et al., 2004; Basavanna et al., 2009; Corsini et al., 2021), suggesting that their elevated protein levels may be associated with the increased virulence of the knockout strain. To validate the quantitative result of proteomics and further explore the effect of SPD_0090 on the virulence of *S. pneumoniae*, the gene expression levels of several virulence factors were determined by RT-qPCR in D39, *D39*Δ*spd0090*, and *D39*Δ*spd0090*^+^. As shown in Figure 7D, the mRNA expression levels of virulence factors *livJ*, *lytC*, and *aliA* of D39Δ*spd0090* were significantly higher than that of D39 (Figure 7D), but their expression in the complemented strain was close to that in the WT (Figure 7E). This result is consistent with the abundance profile of these virulence factors. The increased expression of virulence factors explains why the *spd_0090* gene knockout strain has stronger adhesion and invasion ability to A549 cells than the WT strain.

We also found that two known iron transporters PiuA and SPD_1609 were more abundant in D39Δ*spd0090*. Previous studies have reported that PiuA is an important hemin transporter, and lipoprotein SPD_1609 may be involved in FeCl_3_ or ferrichrome uptake. The alteration of these two genes at the mRNA level was validated by RT-qPCR (Figures 7D,E). The mRNA of *piuA* and *spd*_*1609* in D39Δ*spd0090* was about three and twofold higher than that in the D39, respectively, but there was no significant change in the complement strain (Figure 7E). The upregulation of the iron transports after knockout of the *spd_0090* gene could be to compensate for iron uptake. Importantly, PiuA and SPD_1609 also contribute to the virulence of *S. pneumoniae*^11,14^ which may be another reason for the increased virulence of *D39*Δ*spd0090.*

### GST Pull-Down Identifies the Interaction of SPD_0090 With Virulence Proteins

To further investigate how SPD_0090 affects *S. pneumoniae* virulence, we performed GST pull-down to identify proteins interacting with SPD_0090. A total of 138 interacting proteins were identified by MS after deducting the proteins in the control group. The detailed information on these proteins is listed in Supplementary Table 1. Some cytoplasmic proteins were also identified possibly due to two reasons, i.e., (1) some cytoplasmic proteins bind with proteins that interact with SPD_0090, and (2) the cytoplasmic proteins with high abundance are difficult to be fully removed from the preparation. Among them, three proteins were associated with virulence, including LytC, endopeptidase (PepO), and pneumolysin (Ply). Coincidentally, the elevated protein level of LytC was also detected in D39Δ*spd0090* in quantitative proteomics. We speculated that SPD_0090 interacting with LytC may inhibit LytC function and negatively contribute to bacterial virulence in D39, which requires further validation. In addition, glyceraldehyde-3-phosphate dehydrogenase (Gap), enolase (Eno), and beta-*N*-acetylhexosaminidase (StrH) are associated with bacterial metabolism, which is also consistent with the results identified in the proteomics implying that SPD_0090 affects carbon metabolism in *S. pneumoniae*.

## Discussion

To survive and infect the host, *S. pneumoniae* requires transition metal ions to maintain metabolic homeostasis (Neville et al., 2020). Iron is also essential for the functioning of cellular mechanisms, including enzymatic processes, DNA synthesis, and generating mitochondrial energy (Lopez et al., 2016). However, excessive iron can also be toxic because it catalyzes the production of reactive oxygen species, which can damage lipids, nucleic acids, and proteins (Papanikolaou and Pantopoulos, 2005). Therefore, free iron is rarely available in host cells, not only because of its insolubility but also as a protective mechanism (Cassat and Skaar, 2013). *S. pneumoniae* usually obtain iron by extracting iron-containing molecules (e.g., lactoferrin, transferrin, and hemoglobin) directly from the host (Cassat and Skaar, 2013). This is why bacteria have evolved many iron-transporting proteins, such as the ABC transport proteins PiaABC, PiuABC, PitABC, SPD_1609, and SPD_1590 (Sheldon and Heinrichs, 2015; Yang et al., 2016, 2019; Miao et al., 2018). It is worth noting that they are also important for bacterial virulence. SPD_0090 was highly expressed at the protein level in the triple mutant strain (Δ*piuA/*Δ*piaA/*Δ*pitA*). Therefore, we hypothesized that SPD_0090 is associated with the ability of bacteria to use hemin and with the virulence of *S. pneumoniae*.

In this study, we demonstrated that the lipoprotein SPD_0090 is conserved in a variety of Gram-positive bacteria and is located on the cell membrane. The gene knockout of *spd_0090* resulted in a reduced growth of *S. pneumoniae* under iron-restricted conditions which is partially recovered by supplement with hemin. Meanwhile, the hemin transporter PiuA was obviously highly expressed in the *spd_0090* gene knockout strain to uptake more hemin which would support bacterial growth, but the ferrochrome transporter was not. UV/vis, fluorescence, and EPR spectra revealed that SPD_0090 binds hemin and is associated with hemin transport. Therefore, we suggested that SPD_0090 affects hemin uptake on the cell membrane of *S. pneumoniae.*

Compared to commensal species colonizing the upper respiratory tract, *S. pneumoniae* expresses the most diverse array of sugar transporters and utilization proteins (Buckwalter and King, 2012). More than 30% of the transporter proteins in the *S. pneumoniae* genome were predicted to import carbohydrates as substrates, a higher proportion than any other bacteria (Tettelin et al., 2001). SPD_0090 has been reported in previous studies as a possible galactose ABC transporter protein (Bidossi et al., 2012). Therefore, we cultured knockout strain D39Δ*spd0090* in the medium with galactose and lactose and found a delayed growth relative to D39. ITRAQ quantitative proteomics revealed that both the metabolic tagatose pathway and the Leloir pathway of galactose metabolism had a lower abundance in the D39Δ*spd0090* strain (Figure 7C). In the GST pull-down, we found that SPD_0090 may interact with proteins related to sugar metabolism, such as 6-phospho-β-galactosidase and phosphoenolpyruvate protein PTS. Therefore, the relationship between SPD_0090 and glucose metabolism deserves further study.

More importantly, D39Δ*spd0090* showed enhanced virulence in mice and an improved adhesion and invasion ability to A549 cells compared to the D39 strain. ITRAQ-based proteomics indicated that LytC, AliA, and LivJ virulence factors were highly expressed at protein levels in the knockout strain. Among them, LytC is a cell wall hydrolase, one of the major bacterial components that enables *S. pneumoniae* to lyse non-competent pneumococci (fratricide), enhancing nasopharyngeal colonization and invasiveness (Eldholm et al., 2009; Ramos-Sevillano et al., 2011; Corsini et al., 2021). AliA is an oligopeptide permease on the cell surface and important for bacterial survival in the host and used as an antigenic vaccine to reduce colonization by *S. pneumoniae* (Kerr et al., 2004; Ogunniyi et al., 2012; Nasher et al., 2018a,b; van Beek et al., 2020). LivJ, an amino acid-binding protein of ABC transporter, is responsible for the binding with isoleucine, leucine, and valine (Orihuela et al., 2004; Basavanna et al., 2009). The gene knockout of *livJ* weakened rat meningitis (Molzen et al., 2011). Moreover, subsequent GST pull-down in this study showed that SPD_0090 may interact with virulence factors LytC, PepO, and Ply. We speculated that *D39*Δ*spd0090* exhibited an increased infection ability maintained by upregulating these related virulence factors. Ply is a key pneumococcal virulence factor involved in all phases of pneumococcal disease, including transmission, colonization, and infection (Nishimoto et al., 2020). Hyaluronic acid lyase and plasmin of host cells bind to the surface of pneumococci *via* interaction with enolase (Eno) and glyceraldehyde-3-phosphate dehydrogenase (GAPDH), the bacteria degrade the extracellular matrix (Bergmann et al., 2001; Andre et al., 2017). This disrupts the epithelial barrier and provides a pathway for the paracellular invasion of bacteria (Weiser et al., 2018). *S. pneumoniae* endopeptidase O (PepO) is multifunctional plasminogen and fibronectin-binding protein, facilitating evasion of innate immunity and invasion of host cells (Agarwal et al., 2013). Furthermore, previous reports have shown that mutation of *piuA* and *spd*_*1609* reduced bacterial virulence in bacteremia infection models (Brown et al., 2001a; Whalan et al., 2005; Yang et al., 2019). Both proteins had higher levels in D39Δ*spd0090* than that in D39. Then, in animal experiments, D39Δ*spd0090* infection resulted in increased bacterial counts in lung and nasal, leading to higher mortality in mice than in D39. The combined results suggested that SPD_0090 negatively regulates bacterial virulence.

## Conclusion

**FIGURE 8:**
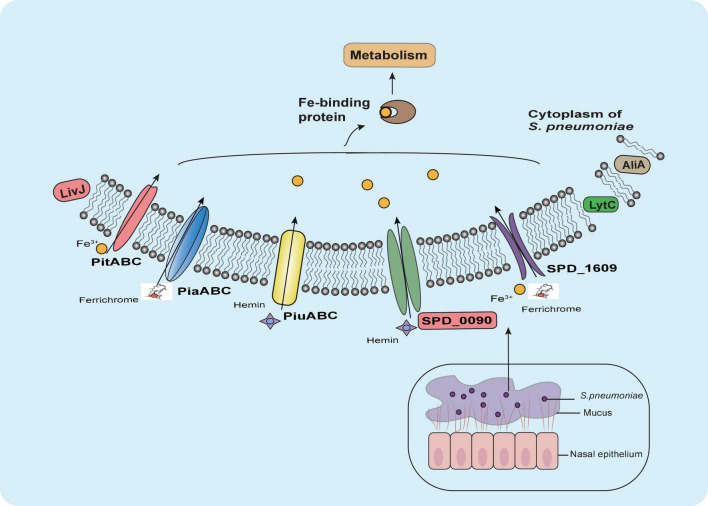
Summary of the role of SPD_0090 in *S. pneumoniae*. SPD_0090, as a lipoprotein of ABC transporter and affects hemin utilization and bacterial carbon metabolism. It also affects the expression of LytC, AliA, and LivJ virulence factors, there by negatively regulating the virulence of *S. pneumoniae.*

Our results suggested that SPD_0090 influences the ability of bacteria to use hemin and negatively regulates bacterial virulence (Figure 8). Our results fill a gap in the functional understanding of SPD_0090 which is highly conserved in Gram-positive bacteria. This finding SPD_0090 will contribute to an in-depth understanding of the pathogenicity of *S. pneumoniae.*

## Data Availability Statement

The data presented in the study are deposited in the PRIDE partner repository, accession number PXD030183.

## Ethics Statement

The animal study was reviewed and approved by Animal Ethics Association of Jinan University.

## Author Contributions

XS, RG, and Q-YH designed the project and revised the manuscript. LC performed the experiments, collected the data, and wrote the manuscript. NL, YD, and JL, performed the experiments. All authors contributed to the article and approved the submitted version.

## Conflict of Interest

The authors declare that the research was conducted in the absence of any commercial or financial relationships that could be construed as a potential conflict of interest.

## Publisher’s Note

All claims expressed in this article are solely those of the authors and do not necessarily represent those of their affiliated organizations, or those of the publisher, the editors and the reviewers. Any product that may be evaluated in this article, or claim that may be made by its manufacturer, is not guaranteed or endorsed by the publisher.

## Footnotes

1
https://www.ncbi.nlm.nih.gov/pmc/

2
https://www.psort.org/psortb/

3
https://services.healthtech.dtu.dk/service.php?SignalP-5.0

4
https://www.ebi.ac.uk/pride/

## Supplementary Material

Supplementary Figure 1 Statistical analysis of bacterial growth curves of bacteria in galactose, glucose, and lactose corresponding to logarithmic growth periods.

Supplementary Table 1 Proteins interacting with SPD_0090 were identified in the GST pull-down assay.

## References

[B1] Agarwal V. Kuchipudi A. Fulde M. Riesbeck K. Bergmann S. Blom A. M. (2013). *Streptococcus pneumoniae* Endopeptidase O (PepO) is a multifunctional plasminogen- and fibronectin-binding protein, facilitating evasion of innate immunity and invasion of host cells. *J. Biol. Chem.* 288 6849–6863. 10.1074/jbc.M112.405530 23341464 PMC3591595

[B2] Andre G. O. Converso T. R. Politano W. R. Ferraz L. F. C. Ribeiro M. L. Leite L. C. C. (2017). Role of *Streptococcus pneumoniae* proteins in evasion of complement-mediated immunity. *Front. Microbiol.* 8:224. 10.3389/fmicb.2017.00224 28265264 PMC5316553

[B3] Armenteros J. A. Tsirigos K. D. Sønderby C. K. Petersen T. N. Winther O. Brunak S. (2019). SignalP 5.0 improves signal peptide predictions using deep neural networks. *Nat. Biotechnol.* 37 420–423. 10.1038/s41587-019-0036-z 30778233

[B4] Basavanna S. Khandavilli S. Yuste J. Cohen J. M. Hosie A. H. F. Webb A. J. (2009). Screening of *Streptococcus pneumoniae* ABC transporter mutants demonstrates that LivJHMGF, a branched-chain amino acid ABC transporter, is necessary for disease pathogenesis. *Infect. Immun.* 77 3412–3423. 10.1128/IAI.01543-08 19470745 PMC2715661

[B5] Bergmann S. Rohde M. Chhatwal G. S. Hammerschmidt S. (2001). α-Enolase of *Streptococcus pneumoniae* is a plasmin(ogen)-binding protein displayed on the bacterial cell surface. *Mol. Microbiol.* 40 1273–1287. 10.1046/j.1365-2958.2001.02448.x 11442827

[B6] Bidossi A. Mulas L. Decorosi F. Colomba L. Ricci S. Pozzi G. (2012). A functional genomics approach to establish the complement of carbohydrate transporters in *Streptococcus pneumoniae*. *PLoS One* 7:e33320. 10.1371/journal.pone.0033320 22428019 PMC3302838

[B7] Bogaert D. de Groot R. Hermans P. W. M. (2004). *Streptococcus pneumoniae* colonisation: the key to pneumococcal disease. *Lancet Infect. Dis.* 4 144–154. 10.1016/S1473-3099(04)00938-7 14998500

[B8] Bordier C. (1981). Phase separation of integral membrane proteins in Triton X-114 solution. *J. Biol. Chem.* 256 1604–1607. 10.1016/S0021-9258(19)69848-0 6257680

[B9] Brown J. S. Gilliland S. M. Holden D. W. (2001a). A *Streptococcus pneumoniae* pathogenicity island encoding an ABC transporter involved in iron uptake and virulence. *Mol. Microbiol.* 40 572–585. 10.1046/j.1365-2958.2001.02414.x 11359564

[B10] Brown J. S. Ogunniyi A. D. Woodrow M. C. Holden D. W. Paton J. C. (2001b). Immunization with components of two iron uptake ABC transporters protects mice against systemic *Streptococcus pneumoniae* infection. *Infect. Immun.* 69 6702–6706. 10.1128/IAI.69.11.6702-6706.2001 11598041 PMC100046

[B11] Brown J. S. Gilliland S. M. Ruiz-Albert J. Holden D. W. (2002). Characterization of pit, a *Streptococcus pneumoniae* iron uptake ABC transporter. *Infect. Immun.* 70 4389–4398. 10.1128/IAI.70.8.4389-4398.2002 12117949 PMC128127

[B12] Buckwalter C. M. King S. J. (2012). Pneumococcal carbohydrate transport: food for thought. *Trends Microbiol.* 20 517–522. 10.1016/j.tim.2012.08.008 22959614 PMC4630977

[B13] Burcham L. R. Hill R. A. Caulkins R. C. Emerson J. P. Nanduri B. Rosch J. W. (2020). *Streptococcus pneumoniae* metal homeostasis alters cellular metabolism†. *Metallomics* 12 1416–1427. 10.1039/d0mt00118j 32676626 PMC7530088

[B14] Cao K. Li N. Wang H. Cao X. He J. Zhang B. (2018). Two zinc-binding domains in the transporter AdcA from *Streptococcus pyogenes* facilitate high-affinity binding and fast transport of zinc. *J. Biol. Chem.* 293 6075–6089. 10.1074/jbc.M117.818997 29491141 PMC5912482

[B15] Carette N. Hagen W. Bertrand L. de Val N. Vertommen D. Roland F. (2006). Optical and EPR spectroscopic studies of demetallation of hemin by L-chain apoferritins. *J. Inorg. Biochem.* 100 1426–1435. 10.1016/j.jinorgbio.2006.03.015 16781777

[B16] Cassat J. E. Skaar E. P. (2013). Iron in infection and immunity. *Cell Host Microbe* 13 509–519. 10.1016/j.chom.2013.04.010 23684303 PMC3676888

[B17] Corsini B. Aguinagalde L. Ruiz S. Domenech M. Yuste J. (2021). Vaccination with LytA, LytC, or Pce of *Streptococcus pneumoniae* protects against sepsis by inducing IgGs that activate the complement system. *Vaccines* 9:186. 10.3390/vaccines9020186 33672306 PMC7926378

[B18] Deutsch E. W. Bandeira N. Sharma V. Perez-Riverol Y. Carver J. J. Kundu D. J. (2020). The ProteomeXchange consortium in 2020: enabling ‘big data’ approaches in proteomics. *Nucleic Acids Res.* 48 D1145–D1152. 10.1093/nar/gkz984 31686107 PMC7145525

[B19] Dong Y. Miao X. Zheng Y. D. Liu J. He Q. Y. Ge R. (2021). Ciprofloxacin-resistant *Staphylococcus aureus* displays enhanced resistance and virulence in iron-restricted conditions. *J. Proteome Res.* 20 2839–2850. 10.1021/acs.jproteome.1c00077 33872026

[B20] Durmort C. Brown J. S. (2015). *Streptococcus pneumoniae* lipoproteins and ABC transporters. *Streptococcus Pneumoniae* 10 181–206. 10.1016/B978-0-12-410530-0.00010-7

[B21] Eldholm V. Johnsborg O. Haugen K. Ohnstad H. S. Håvarstein L. S. (2009). Fratricide in *Streptococcus pneumoniae*: contributions and role of the cell wall hydrolases CbpD, LytA and LytC. *Microbiology* 155 2223–2234. 10.1099/mic.0.026328-0 19389766

[B22] Engholm D. H. Kilian M. Goodsell D. S. Andersen E. S. Kjærgaard R. S. (2017). A visual review of the human pathogen *Streptococcus pneumoniae*. *FEMS Microbiol. Rev.* 41 854–879. 10.1093/femsre/fux037 29029129

[B23] Gan C. S. Chong P. K. Pham T. K. Wright P. C. (2007). Technical, experimental, and biological variations in isobaric tags for relative and absolute quantitation (iTRAQ). *J. Proteome Res.* 6 821–827. 10.1021/pr060474i 17269738

[B24] Gupta R. Shah P. Swiatlo E. (2009). Differential gene expression in *Streptococcus pneumoniae* in response to various iron sources. *Microb. Pathog.* 47 101–109. 10.1016/j.micpath.2009.05.003 19464356

[B25] Honsa E. Johnson M. Rosch J. (2013). The roles of transition metals in the physiology and pathogenesis of *Streptococcus pneumoniae*. *Front. Cell. Infect. Microbiol.* 3:92. 10.3389/fcimb.2013.00092 24364001 PMC3849628

[B26] Jomaa M. Yuste J. Paton James C. Jones C. Dougan G. Brown Jeremy S. (2005). Antibodies to the iron uptake ABC transporter lipoproteins PiaA and PiuA promote opsonophagocytosis of *Streptococcus pneumoniae*. *Infect. Immun.* 73 6852–6859. 10.1128/IAI.73.10.6852-6859.2005 16177364 PMC1230898

[B27] Kadioglu A. Weiser J. N. Paton J. C. Andrew P. W. (2008). The role of *Streptococcus pneumoniae* virulence factors in host respiratory colonization and disease. *Nat. Rev. Microbiol.* 6 288–301. 10.1038/nrmicro1871 18340341

[B28] Kerr A. R. Adrian P. V. Estevão S. Groot R. D. Alloing G. Claverys J.-P. (2004). The Ami-AliA/AliB permease of *Streptococcus pneumoniae* is involved in nasopharyngeal colonization but not in invasive disease. *Infect. Immun.* 72 3902–3906. 10.1128/IAI.72.7.3902-3906.2004 15213133 PMC427416

[B29] Khandavilli S. Homer K. A. Yuste J. Basavanna S. Mitchell T. Brown J. S. (2008). Maturation of *Streptococcus pneumoniae* lipoproteins by a type II signal peptidase is required for ABC transporter function and full virulence. *Mol. Microbiol.* 67 541–557. 10.1111/j.1365-2958.2007.06065.x 18086214 PMC2228790

[B30] Lopez A. Cacoub P. Macdougall I. C. Peyrin-Biroulet L. (2016). Iron deficiency anaemia. *Lancet* 387 907–916. 10.1016/S0140-6736(15)60865-0 26314490

[B31] Lopez C. A. Skaar E. P. (2018). The impact of dietary transition metals on host-bacterial interactions. *Cell Host Microbe* 23 737–748. 10.1016/j.chom.2018.05.008 29902439 PMC6007885

[B32] Lu J. Bitoun J. P. Tan G. Wang W. Min W. Ding H. (2010). Iron-binding activity of human iron-sulfur cluster assembly protein hIscA1. *Biochem. J.* 428 125–131. 10.1042/bj20100122 20302570 PMC2878720

[B33] Miao X. He J. Zhang L. Zhao X. Ge R. He Q. Y. (2018). A novel iron transporter SPD_1590 in *Streptococcus pneumoniae* contributing to bacterial virulence properties. *Front. Microbiol.* 9:1624. 10.3389/fmicb.2018.01624 30079056 PMC6062600

[B34] Molzen T. E. Burghout P. Bootsma H. J. Brandt C. T. van der Gaast-de Jongh C. E. Eleveld M. J. (2011). Genome-wide identification of *Streptococcus pneumoniae* genes essential for bacterial replication during experimental meningitis. *Infect. Immun.* 79 288–297. 10.1128/IAI.00631-10 21041497 PMC3019918

[B35] Nasher F. Aguilar F. Aebi S. Hermans P. W. M. Heller M. Hathaway L. J. (2018a). Peptide ligands of AmiA, AliA, and AliB proteins determine pneumococcal phenotype. *Front. Microbiol.* 9:3013. 10.3389/fmicb.2018.03013 30568648 PMC6290326

[B36] Nasher F. Heller M. Hathaway L. J. (2018b). *Streptococcus pneumoniae* proteins AmiA, AliA, and AliB Bind peptides found in ribosomal proteins of other bacterial species. *Front. Microbiol.* 8:2688. 10.3389/fmicb.2017.02688 29379482 PMC5775242

[B37] Neville S. L. Eijkelkamp B. A. Lothian A. Paton J. C. Roberts B. R. Rosch J. W. (2020). Cadmium stress dictates central carbon flux and alters membrane composition in *Streptococcus pneumoniae*. *Commun. Biol.* 3:694. 10.1038/s42003-020-01417-y 33214631 PMC7678824

[B38] Nguyen M. T. Götz F. (2016). Lipoproteins of Gram-positive bacteria: key players in the immune response and virulence. *Microbiol. Mol. Biol. Rev.* 80 891–903. 10.1128/MMBR.00028-16 27512100 PMC4981669

[B39] Nishimoto A. T. Rosch J. W. Tuomanen E. I. (2020). Pneumolysin: pathogenesis and therapeutic target. *Front. Microbiol.* 11:1543. 10.3389/fmicb.2020.01543 32714314 PMC7343714

[B40] Ogunniyi A. D. Mahdi L. K. Trappetti C. Verhoeven N. Mermans D. Hoek M. B. V. D. (2012). Identification of genes that contribute to the pathogenesis of invasive pneumococcal disease by *in vivo* transcriptomic analysis. *Infect. Immun.* 80 3268–3278. 10.1128/IAI.00295-12 22778095 PMC3418729

[B41] Orihuela C. J. Radin J. N. Sublett J. E. Gao G. Kaushal D. Tuomanen E. I. (2004). Microarray analysis of pneumococcal gene expression during invasive disease. *Infect. Immun.* 72 5582–5596. 10.1128/IAI.72.10.5582-5596.2004 15385455 PMC517545

[B42] Palmer L. D. Skaar E. P. (2016). Transition metals and virulence in bacteria. *Annu. Rev. Genet.* 50 67–91. 10.1146/annurev-genet-120215-035146 27617971 PMC5125913

[B43] Papanikolaou G. Pantopoulos K. (2005). Iron metabolism and toxicity. *Toxicol. Appl. Pharmacol.* 202 199–211. 10.1016/j.taap.2004.06.021 15629195

[B44] Payne S. M. Neilands I. (1988). Iron and virulence in the family *Enterobacteriaceae*. *Crit. Rev. Microbiol.* 16 81–111. 10.3109/10408418809104468 3067977

[B45] Quin L. R. Onwubiko C. Moore Q. C. Mills M. F. McDaniel L. S. Carmicle S. (2007). Factor H binding to PspC of *Streptococcus pneumoniae* increases adherence to human cell lines *in vitro* and enhances invasion of mouse lungs *in vivo*. *Infect. Immun.* 75 4082–4087. 10.1128/iai.00474-07 17562771 PMC1952001

[B46] Ramos-Sevillano E. Moscoso M. García P. García E. Yuste J. (2011). Nasopharyngeal colonization and invasive disease are enhanced by the cell wall hydrolases LytB and LytC of *Streptococcus pneumoniae*. *PLoS One* 6:e23626. 10.1371/journal.pone.0023626 21886805 PMC3160309

[B47] Robb M. Hobbs J. K. Woodiga S. A. Shapiro-Ward S. Suits M. D. L. McGregor N. (2017). Molecular characterization of N-glycan degradation and transport in *Streptococcus pneumoniae* and its contribution to virulence. *PLoS Pathog.* 13:e1006090. 10.1371/journal.ppat.1006090 28056108 PMC5215778

[B48] Shannon P. Markiel A. Ozier O. Baliga N. S. Wang J. T. Ramage D. (2003). Cytoscape: a software environment for integrated models of biomolecular interaction networks. *Genome Res.* 13 2498–2504. 10.1101/gr.1239303 14597658 PMC403769

[B49] Sheldon J. R. Heinrichs D. E. (2015). Recent developments in understanding the iron acquisition strategies of gram positive pathogens. *FEMS Microbiol. Rev.* 39 592–630. 10.1093/femsre/fuv009 25862688

[B50] Tettelin H. Nelson K. E. Paulsen I. T. Eisen J. A. Read T. D. Peterson S. (2001). Complete genome sequence of a virulent isolate of *Streptococcus pneumoniae*. *Science* 293 498–506. 10.1126/science.1061217 11463916

[B51] van Beek L. F. Surmann K. van den Berg van Saparoea H. B. Houben D. Jong W. S. P. Hentschker C. (2020). Exploring metal availability in the natural niche of *Streptococcus pneumoniae* to discover potential vaccine antigens. *Virulence* 11 1310–1328. 10.1080/21505594.2020.1825908 33017224 PMC7550026

[B52] Wach A. (1996). PCR-synthesis of marker cassettes with long flanking homology regions for gene disruptions in *S. cerevisiae*. *Yeast* 12 259–265. 8904338 10.1002/(SICI)1097-0061(19960315)12:3%3C259::AID-YEA901%3E3.0.CO;2-C

[B53] Weight C. M. Jochems S. P. Adler H. Ferreira D. M. Brown J. S. Heyderman R. S. (2021). Insights into the effects of mucosal epithelial and innate immune dysfunction in older people on host interactions with *Streptococcus pneumoniae*. *Front. Cell. Infect. Microbiol.* 11:651474. 10.3389/fcimb.2021.651474 34113578 PMC8185287

[B54] Weiser J. N. Ferreira D. M. Paton J. C. (2018). *Streptococcus pneumoniae*: transmission, colonization and invasion. *Nat. Rev. Microbiol.* 16 355–367. 10.1038/s41579-018-0001-8 29599457 PMC5949087

[B55] Whalan R. H. Funnell S. G. Bowler L. D. Hudson M. J. Robinson A. Dowson C. G. (2005). PiuA and PiaA, iron uptake lipoproteins of *Streptococcus pneumoniae*, elicit serotype independent antibody responses following human pneumococcal septicaemia. *FEMS Immunol. Med. Microbiol.* 43 73–80. 10.1016/j.femsim.2004.07.010 15607639

[B56] Yang X. Y. He K. Du G. Wu X. Yu G. Pan Y. (2016). Integrated translatomics with proteomics to identify novel iron-transporting proteins in *Streptococcus pneumoniae*. *Front. Microbiol.* 7:78. 10.3389/fmicb.2016.00078 26870030 PMC4738293

[B57] Yang X. Y. Li N. Xu J. Y. Sun X. He Q. Y. (2019). Lipoprotein SPD_1609 of *Streptococcus pneumoniae* promotes adherence and invasion to epithelial cells contributing to bacterial virulence. *Front. Microbiol.* 10:1769. 10.3389/fmicb.2019.01769 31417540 PMC6682666

[B58] Yu N. Y. Wagner J. R. Laird M. R. Melli G. Rey S. Lo R. (2010). PSORTb 3.0: improved protein subcellular localization prediction with refined localization subcategories and predictive capabilities for all prokaryotes. *Bioinformatics* 26 1608–1615. 10.1093/bioinformatics/btq249 20472543 PMC2887053

[B59] Zheng Y.-D. Pan Y. He K. Li N. Yang D. Du G.-F. (2021). SPD_1495 contributes to capsular polysaccharide synthesis and virulence in *Streptococcus pneumoniae*. *mSystems* 5:e00025-20. 10.1128/mSystems.00025-20 32098834 PMC7043342

[B60] Zhou Q. Yan L. Xu B. Wang X. E. Sun X. Han N. (2021). Screening of the HBx transactivation domain interacting proteins and the function of interactor Pin1 in HBV replication. *Sci. Rep.* 11:14176. 10.1038/s41598-021-93584-z 34238995 PMC8266847

