# Supplementary figures and images for: SPD_0090 Negatively Contributes to Virulence of Streptococcus pneumoniae
Source: Front Microbiol. 2022 Jun 13;13:896896. doi: 10.3389/fmicb.2022.896896 (PMC9234739; doi:10.3389/fmicb.2022.896896)

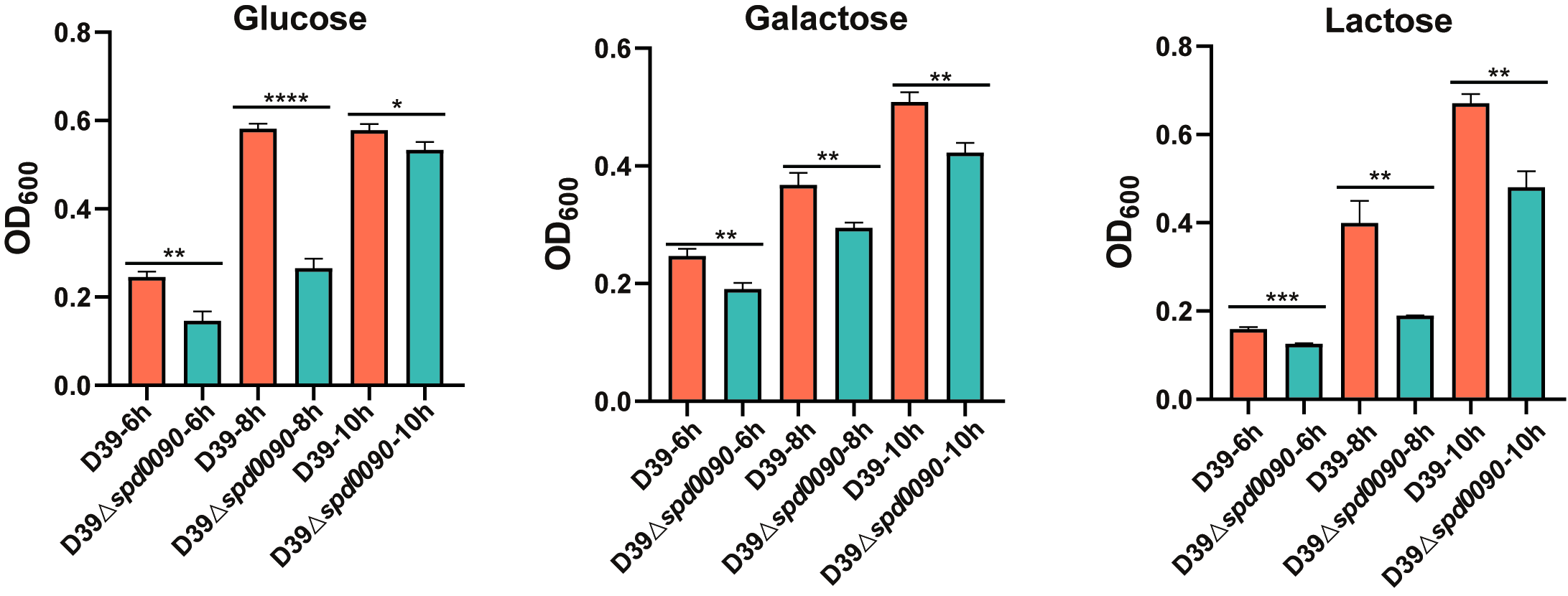

Supplement: Supplementary Figure 1 — Statistical analysis of bacterial growth curves of bacteria in galactose, glucose, and lactose corresponding to logarithmic growth periods. [file Image_1.TIF]
