# Supplementary material for: SPD_0090 Negatively Contributes to Virulence of Streptococcus pneumoniae
Source: Front Microbiol. 2022 Jun 13;13:896896. doi: 10.3389/fmicb.2022.896896 (PMC9234739; doi:10.3389/fmicb.2022.896896)
Supplement: Supplementary Table 1 — Proteins interacting with SPD_0090 were identified in the GST pull-down assay. [file Table_1.DOCX]

Table S1. Proteins interacting with of SPD_0090 identified in GST pull down assay. The localizations of these proteins were analyzed using PSORTb version 3.0.3.

| **Accessions** | **Protein names** | **Gene names** |
| --- | --- | --- |
| **Membrane** | |  |
| A0A0H2ZQB9 | Choline transporter (Glycine betaine transport system permease protein) | *proWX* |
| A0A0H2ZM11 | Signal recognition particle protein | *ffh* |
| A0A0H2ZRX9 | ABC transporter, ATP-binding/permease protein | *spd1902* |
| A0A0H2ZPN2 | Membrane protein, putative | *spd1867* |
| A0A0H2ZRZ4 | Capsular polysaccharide biosynthesis protein | *capD* |
| A0A0H2ZMV7 | Sugar ABC transporter, ATP-binding protein | *spd0740* |
| A0A0H2ZQ94 | PTS system, IIABC components | *exp5* |
| A0A0H2ZLY2 | Glycerol uptake facilitator protein, putative | *spd1320* |
| Q04IS6 | UPF0154 protein SPD_1662 | *spd1662* |
| A0A0H2ZRM1 | Oligopeptide ABC transporter, permease protein AmiC | *amiC* |
| A0A0H2ZMV8 | Uracil-xanthine permease | *uraA* |
| A0A0H2ZNS9 | Amino acid ABC transporter, amino acid-binding protein/permease protein | *spd0412* |
| A0A0H2ZR55 | Uncharacterized protein | *spd1380* |
| Q04KB7 | Elongation factor 4 | *lepA* |
| A0A0H2ZQE6 | Amino acid ABC transporter, ATP-binding protein | *spd1289* |
| Q04JW0 | Spermidine/putrescine import ATP-binding protein PotA | *potA* |
| A0A0H2ZN94 | PTS system, fructose specific IIABC components | *spd0773* |
| A0A0H2ZMA2 | ATP-dependent zinc metalloprotease FtsH | *ftsH* |
| **Cytoplasmic** |  |  |
| A0A0H2ZQ35 | Endopeptidase O | *pepO* |
| A0A0H2ZNS0 | Glyceraldehyde-3-phosphate dehydrogenase | *gap* |
| Q04KG2 | Enolase | *eno* |
| A0A0H2ZQ14 | Uncharacterized protein | *spd1416* |
| A0A0H2ZLJ0 | Cof family protein/peptidyl-prolyl cis-trans isomerase, cyclophilin type | *spd1367* |
| A0A0H2ZNT2 | Inosine-5'-monophosphate dehydrogenase | *guaB* |
| A0A0H2ZQQ0 | Threonine synthase | *thrC* |
| A0A0H2ZRA0 | Lysine decarboxylase | *cad* |
| Q04MC1 | UPF0371 protein SPD_0310 | *spd0310* |
| A0A0H2ZM51 | Nicotinate phosphoribosyltransferase | *pncB* |
| A0A0H2ZNQ8 | Type I restriction-modification system, M subunit | *hsdM* |
| Q04IQ3 | 60 kDa chaperonin | *groL* |
| A0A0H2ZM63 | Type I restriction-modification system, S subunit | *hsdS* |
| Q04J36 | Ribonuclease Y | *rny* |
| Q04I94 | Arginine--tRNA ligase | *argS* |
| A0A0H2ZLU9 | Transketolase | *tkt* |
| A0A0H2ZPV8 | DNA topoisomerase 1 | *topA* |
| A0A0H2ZNQ5 | Ribonuclease R | *rnr* |
| A0A0H2ZMD1 | DNA topoisomerase 4 subunit A | *parC* |
| A0A0H2ZPQ1 | Type II restriction endonuclease, putative | *spd1079* |
| A0A0H2ZNN6 | Transcription-repair-coupling factor | *mfd* |
| A0A0H2ZPZ8 | 6-phospho-beta-galactosidase | *lacG-2* |
| A0A0H2ZP45 | Glutamine--fructose-6-phosphate aminotransferase [isomerizing] | *glmS* |
| A0A0H2ZMY1 | Phosphoenolpyruvate-protein phosphotransferase | *ptsI* |
| Q04K99 | L-lactate dehydrogenase | *ldh* |
| A0A0H2ZNC9 | Glucose-6-phosphate 1-dehydrogenase | *zwf* |
| Q04I37 | 50S ribosomal protein L32 | *rpmF* |
| Q04JT6 | 30S ribosomal protein S21 | *rpsU* |
| Q04KW8 | 50S ribosomal protein L35 | *rpmI* |
| Q04JL0 | 30S ribosomal protein S18 | *rpsR* |
| Q04MM3 | 30S ribosomal protein S14 | *rpsN* |
| Q04LD0 | 30S ribosomal protein S16 | *rpsP* |
| Q04KI2 | 50S ribosomal protein L27 | *rpmA* |
| Q04JE2 | 30S ribosomal protein S15 | *rpsO* |
| Q04MN2 | 30S ribosomal protein S19 | *rpsS* |
| Q04MN4 | 50S ribosomal protein L23 | *rplW* |
| Q04MM5 | 50S ribosomal protein L24 | *rplX* |
| Q04MN7 | 30S ribosomal protein S10 | *rpsJ* |
| A0A0H2ZR51 | Cell division protein ZapA | *spd0369* |
| Q04MN1 | 50S ribosomal protein L22 | *rplV* |
| Q04JZ4 | 50S ribosomal protein L7/L12 | *rplL* |
| Q04M95 | Cell cycle protein GpsB | *gpsB* |
| Q04MM0 | 50S ribosomal protein L18 | *rplR* |
| Q04K32 | 50S ribosomal protein L19 | *rplS* |
| A0A0H2ZMP4 | Uncharacterized protein | *spd0680* |
| Q04MH9 | 30S ribosomal protein S12 | *rpsL* |
| Q04ML9 | 30S ribosomal protein S5 | *rpsE* |
| Q04K45 | Bifunctional protein PyrR | *pyrR* |
| A0A0H2ZNN3 | General stress protein 24, putative | *spd1590* |
| A0A0H2ZM67 | Uracil phosphoribosyltransferase | *upp* |
| Q04HW3 | 30S ribosomal protein S2 | *rpsB* |
| Q04JL5 | Probable manganese-dependent inorganic pyrophosphatase | *ppaC* |
| A0A0H2ZNR1 | Transcriptional regulator PlcR, putative | *spd1786* |
| Q04J98 | D-alanine--D-alanine ligase | *ddl* |
| Q04LZ5 | Phosphoglycerate kinase | *pgk* |
| A0A0H2ZL13 | Transcription termination/antitermination protein NusA | *nusA* |
| A0A0H2ZLT2 | Ribosomal protein S1 | *rpsA* |
| A0A0H2ZMP2 | Riboflavin biosynthesis protein RibBA | *ribB* |
| Q04KZ2 | Gamma-glutamyl phosphate reductase | *proA* |
| A0A0H2ZQS7 | UDP-N-acetylglucosamine 1-carboxyvinyltransferase | *murA-2* |
| A0A0H2ZMP6 | FeS assembly protein SufD | *sufD* |
| Q04M73 | Trigger factor | *tig* |
| A0A0H2ZP62 | Peptidase, U32 family protein | *spd1258* |
| Q04KK7 | GTPase Obg | *obg* |
| A0A0H2ZPT5 | Cell division protein FtsA | *ftsA* |
| A0A0H2ZPB4 | NADH oxidase | *nox* |
| A0A0H2ZNI4 | Glutamine synthetase, type I | *glnA* |
| A0A0H2ZMN4 | Dipeptidase PepV | *pepV* |
| A0A0H2ZPR8 | DEAD-box ATP-dependent RNA helicase CshB | *cshB* |
| A0A0H2ZQD1 | Cysteine--tRNA ligase | *cysS* |
| Q04JK7 | Asparagine--tRNA ligase | *asnS* |
| Q04N63 | Chromosomal replication initiator protein DnaA | *dnaA* |
| Q04M42 | Aspartyl/glutamyl-tRNA (Asn/Gln) amidotransferase subunit B | *gatB* |
| Q04MI4 | Proline--tRNA ligase | *proS* |
| Q04JD7 | Threonine--tRNA ligase | *thrS* |
| A0A0H2ZN13 | Aminopeptidase N | *pepN* |
| Q04JW5 | Alanine--tRNA ligase | *alaS* |
| A0A0H2ZPZ1 | Alcohol dehydrogenase, zinc-containing | *spd0265* |
| A0A0H2ZRI0 | Alcohol dehydrogenase, zinc-containing | *spd1865* |
| Q04KU2 | Bifunctional protein GlmU | *glmU* |
| A0A0H2ZNN4 | Glyceraldehyde-3-phosphate dehydrogenase, NADP-dependent | *gapN* |
| A0A0H2ZMB7 | Non-heme iron-containing ferritin | *spd1402* |
| A0A0H2ZM97 | Pseudouridine synthase | *rluB* |
| A0A0H2ZN23 | Capsular polysaccharide biosynthesis protein, putative | *spd1619* |
| A0A0H2ZP94 | Saccharopine dehydrogenase | *lys1* |
| Q04N48 | Adenylosuccinate synthetase | *purA* |
| A0A0H2ZP60 | NOL1/NOP2/sun family protein | *spd1233* |
| A0A0H2ZR44 | Aminopeptidase C | *pepC* |
| A0A0H2ZN51 | UDP-N-acetylmuramoyl-tripeptide--D-alanyl-D-alanine ligase | *murF* |
| Q04JI8 | Phosphoglucosamine mutase | *glmM* |
| A0A0H2ZNI3 | Acetyl-CoA carboxylase, biotin carboxylase | *accC`* |
| Q04M41 | Glutamyl-tRNA (Gln) amidotransferase subunit A | *gatA* |
| A0A0H2ZLA5 | 6-phosphogluconate dehydrogenase, decarboxylating | *gnd* |
| A0A0H2ZQT2 | 4-alpha-glucanotransferase | *malQ* |
| A0A0H2ZRU3 | ABC transporter, ATP-binding protein | *spd1137* |
| A0A0H2ZMB9 | ATP-dependent Clp protease, ATP-binding subunit | *clpL* |
| Q04M67 | Endonuclease MutS2 | *mutS2* |
| Q04K48 | Carbamoyl-phosphate synthase large chain | *carB* |
| A0A0H2ZNG5 | Chromosome partition protein Smc | *smc* |
| **Extracellular** |  |  |
| A0A0H2ZNC0 | 1,4-beta-N-acetylmuramidase, putative | *lytC* |
| Q04IN8 | Pneumolysin | *ply* |
| **Cell wall** |  |  |
| A0A0H2ZQY4 | Beta-N-acetylhexosaminidase | *strH* |
| A0A0H2ZM44 | Oligopeptide ABC transporter,oligopeptide-binding protein | *spd1170* |
| A0A0H2ZM01 | Cell wall surface anchor family protein | *spd0080* |
| A0A0H2ZM17 | Cell wall surface anchor family protein | *spd0335* |
| **Unknown** |  |  |
| A0A0H2ZPG7 | Mid-cell-anchored protein Z | *mapZ* |
| A0A0H2ZMF0 | Type I restriction-modification system, M subunit, putative | *spd0782* |
| A0A0H2ZP20 | Preprotein translocase, YajC subunit | *yajC-2* |
| Q04KI4 | 50S ribosomal protein L21 | *rplU* |
| A0A0H2ZMP5 | Uncharacterized protein | *spd1928* |
| A0A0H2ZQB3 | Bacterocin transport accessory protein | *bta* |
| A0A0H2ZLI7 | Uncharacterized protein | *spd0978* |
| A0A0H2ZNW1 | Septum formation initiator, putative | *spd0008* |
| Q04JC5 | D-aminoacyl-tRNA deacylase | *dtd* |
| Q04MN5 | 50S ribosomal protein L4 | *rplD* |
| A0A0H2ZNE0 | Cell division protein FtsZ | *ftsZ* |
| A0A0H2ZQ70 | Oxidoreductase, putative | *spd1302* |
| A0A0H2ZLQ1 | Endolytic murein transglycosylase | *mltG* |
| Q04JC5 | D-aminoacyl-tRNA deacylase | *dtd* |
| A0A0H2ZNJ9 | Uncharacterized protein | *spd1072* |

Yu, N.Y., Wagner, J.R., Laird, M.R., Melli, G., Rey, S., Lo, R., et al. (2010). PSORTb 3.0: improved protein subcellular localization prediction with refined localization subcategories and predictive capabilities for all prokaryotes. *Bioinformatics.* 26(13)**,** 1608-1615. doi: 10.1093/bioinformatics/btq249.
